# Golden biomass: unlocking the potential of neglected potato (*Solanum tuberosum* L.) side-streams as sources of bioactive compounds

**DOI:** 10.3389/fpls.2026.1957940

**Published:** 2026-10-05

**Authors:** Marília Bueno da Silva, Björn Thiele, Björn Usadel, Franziska Genzel, Anika Wiese-Klinkenberg

**Affiliations:** 1 Institute of Bio- and Geosciences (IBG-4: Bioinformatics), CEPLAS, Forschungszentrum Jülich GmbH, Jülich, Germany; 2 Bioeconomy Science Center (BioSC), Forschungszentrum Jülich GmbH, Jülich, Germany; 3 Institute of Bio- and Geosciences (IBG-2: Plant Sciences), Forschungszentrum Jülich GmbH, Jülich, Germany; 4 Institute of Bio- and Geosciences (IBG-3: Agrosphere), Forschungszentrum Jülich GmbH, Jülich, Germany; 5 Faculty of Mathematics and Natural Sciences, CEPLAS, Institute for Biological Data Science, Heinrich Heine University Düsseldorf, Düsseldorf, Germany

**Keywords:** above-ground biomass, by-products, metabolomics, potato glycoalkaloids, transcriptomics

## Abstract

**Background:**

Potato flowers and berries are largely unused agricultural side-streams that may provide bioactive compounds for pharmaceutical and plant protection applications in a circular bioeconomy. This study evaluated their potential as sources of secondary metabolites and assessed whether premature harvesting affects final tuber yield.

**Methods:**

Field trials with two potato cultivars were combined with targeted HPLC-MS/MS analysis, untargeted metabolomics, and RNA-Seq analysis of flowers and developing berries.

**Results:**

Berries produced substantially more biomass than flowers, whereas flowers generally contained higher concentrations of total glycoalkaloids (calculated as the sum of a-solanine and a-chaconine). This reached 393.95 mg/100 g FW in ‘Quarta’ flowers compared to 70.92 mg/100 g FW in berries, with the concentration of a-chaconine up to 3.33-fold higher than that of a-solanine. Untargeted metabolomics identified glycoalkaloids, phenolic compounds, and other secondary metabolites, with peak intensities of rutin and some others declining from ~1Å~10^9^ to ~1Å~10^5^ during berry development. Transcriptomic analysis showed that tissue and developmental stage had a substantially greater influence on gene expression patterns than cultivar. Genes associated with phenylpropanoid, terpenoid and steroid-related pathways were differentially expressed, with overall stronger upregulation in flowers than in small and big berries. Premature flower harvest did not reduce tuber yield, whereas berry harvest reduced yield for one cultivar in one field experiment.

**Conclusion:**

Overall, flowers and berries harvested as side streams in the potato production can be a good source for glycoalkaloids and possibly further metabolites of use for pharmaceutical and plant protection applications. Flowers appear particularly suitable for recovering high-concentration bioactive compounds, whereas berries offer advantages in biomass availability, harvesting, and storage.

## 1 Introduction

Potato (*Solanum tuberosum* L.) is a globally important food crop first domesticated in the Andean region ~6000 years ago (Spooner, 2013). The tubers (underground biomass) are a staple food in both eastern and western cultures, contributing significantly to food security by offering high yield potential and high-quality nutrition (Food and Agriculture Organization of the United Nations, 2024; Devaux et al., 2021). The tubers are the edible and marketable part of potato plants, rich in macronutrients such as starch and protein, dietary fiber, and micronutrients such as vitamins B and C, potassium, calcium and magnesium (Singh et al., 2014; Beals, 2019). The non-edible aboveground biomass (stems, leaves, flowers and berries) constitutes the side-streams or by-products of potato cultivation (Bueno da Silva et al., 2024). Leaves provide a source of assimilates for the tubers (Gu et al., 2024; Chen et al., 2025; Mukiibi et al., 2025), whereas flowers and berries are reproductive organs and may act as competing sinks (Almekinders and Struik, 1996). This biomass currently has limited commercial value in the potato sector, and is often left in the field after tuber harvest or burned.

In addition to its nutritional composition, potato plants also contain many bioactive phytochemicals (Singh et al., 2014). These include phenolic compounds such as coumarins, anthocyanins, chlorogenic acid, *p*-coumaric acids, kaempferol, naringenin and rutin (Ezekiel et al., 2013); carotenoids such as lutein, zeaxanthin and violaxanthin; and steroidal glycoalkaloids, primarily α-solanine and α-chaconine (Friedman et al., 2018; Beals, 2019; Bueno da Silva et al., 2024). The phytochemical composition of potato tissues may vary according to genotype, agronomic practices, tissue type and food processing (Ezekiel et al., 2013; Singh et al., 2014). For example, purple-flesh cultivars often contain higher concentrations of anthocyanins than white-fleshed cultivars (Vaitkevičienė et al., 2020), whereas cooking can reduce glycoalkaloid concentrations to different extents (Rytel et al., 2005; Lachman et al., 2013; Martínez-García et al., 2024) and alter the levels of phenolic compounds (Lachman et al., 2013; Fang et al., 2022).

The tuber-processing market is expected to grow by 4.9% annually, reaching USD 60.08 billion by 2031 (Hu et al., 2025). The production of French fries and potato chips generates waste streams comprising peels, pulp and offcuts, which account for 12–20% of the total processed volume (Chauhan et al., 2023). In addition, starch extraction generates substantial quantities of potato juice (Kowalczewski et al., 2022). However, these industrial side-streams can be valorized through a range of applications, including animal feeding (Ncobela et al., 2017), the production of biogas (Sanaei-Moghadam et al., 2014) and biofuel (Liang and McDonald, 2014), and the extraction of bioactive molecules (Friedman et al., 2018; Kowalczewski et al., 2022). Most research on the potential of potato side-streams has therefore focused on such processing waste as a means to support potato production in a circular economy (Pęksa and Miedzianka, 2021; Chauhan et al., 2023; Khanal et al., 2024) but has largely ignored agricultural waste. Farmers tend to remove or destroy aboveground potato biomass to reduce disease pressure and promote tuber maturation, but a small number of studies have examined its potential for industrial valorization (Kempenaar and Struik, 2007; Li et al., 2022). For example, Gupta et al. (2016) found that powdered potato leaves and stems had rough surfaces and high adsorption capacity, enabling their potential use as adsorbents to remove dye pollutants such as methylene blue and malachite green. Chávez and Li (2025) assessed the water content of aboveground potato biomass to evaluate its potential for thermochemical conversion. Beyond their favorable physical characteristics, potato agricultural side-streams may also serve as sources of phytochemicals for industrial applications, although little is known about the metabolic content of aboveground biomass (Bueno da Silva et al., 2024).

Here, we evaluated the role of potato flowers and berries as a source of bioactive compounds by investigating (1) the feasibility of early harvest and its effect on tuber yield, (2) the distribution of glycoalkaloids and phenolic compounds among different tissues and cultivars, and (3) the profiles of metabolites and gene expression to identify valuable compounds for extraction and corresponding biosynthetic genes, thereby supporting the valorization of these side-streams in a circular bioeconomy.

## 2 Materials and methods

### 2.1 Field trials

To study how the early harvest of potato flowers and berries affects final tuber yield, we conducted two field trials with cultivars ‘Quarta’ and ‘Granola’ in the years 2022 and 2023 at Campus Klein-Altendorf (CKA), University of Bonn, Rheinbach, Germany. In May to September 2022, potato plants were cultivated on a total area of 127.5 m^2^ as a randomized block design (four blocks), with six treatment rows (target cultivars) and two extra border rows left and right from the blocks with red-skinned potato cultivar (cv. ‘Foxton’). In May to October 2023, the total area was increased to 346.3 m^2^ with six randomized blocks, nine treatment rows (including an additional cultivar ‘Innovator’, data not shown), and extra borders around the complete block (cv. ‘Laura’) and one further extra row as a filler (cv. ‘Gala’). The extra border rows were planted to eliminate border effects and were not used in any evaluation. The field designs are illustrated in **Supplementary Figures S1 and S2**.

In both experiments, potatoes were sown with a spacing of 0.75 m between rows and 0.33 m between plants, with 3.3 m between blocks, each row containing 10 plants (**Figures S1 and S2**). We set up the following treatments: Control – no harvest of flowers or berries; Flower – harvest of flowers before the harvest of tubers, BBCH 635 (full flowering, 50% of flowers in the third inflorescence open) (Hack et al., 1993); Berry – harvest of berries before the harvest of tubers, BBCH 721 (10% of berries in the second fructification have reached full size) (Hack et al., 1993).

By-products were harvested manually and immediately frozen in liquid nitrogen. Pest and weed controls were implemented according to standard local agronomic schedules. At the end of the crop cycle, tubers of all cultivars were mechanically harvested, and the calculation of average tuber yield (kg/ha) considered all harvested tubers, regardless of any coloration or damage. By-product yields (kg/ha) were also calculated for both years.

### 2.2 HPLC-MS/MS quantification of secondary metabolites

#### 2.2.1 Solvents and reference substances

For HPLC-MS/MS analysis, HPLC-grade water and acetonitrile (ACN, CAS 75-05-8) were purchased from Carl Roth (Karlsruhe, Germany), and methanol (CAS 67-56-1) was obtained from VWR International (Darmstadt, Germany). The reference glycoalkaloids solanidine (C_27_H_43_NO, CAS 80-78-4), solasodine (C_27_H_43_NO_2_, CAS 126-17-0), α-solanine (C_45_H_73_NO_15_, CAS 20562-02-1), α-chaconine (C_45_H_73_NO_14_, CAS 20562-03-2), and α-solamargine (C_45_H_73_NO_15_, CAS 20311-51-7) were purchased from PhytoLab (Vestenbergsgreuth, Germany). We also purchased the following reference phenolic compounds: coniferin (C_16_H_22_O_8_, CAS 531-29-3) from BLDpharm (Shanghai, China), metabolite P (2,6-dihydroxy-2-[(4-hydroxyphenyl)methyl]-4-[(2S,3R,4S,5S,6R)-3,4,5-trihydroxy-6-(hydroxymethyl)oxan-2-yl]oxy-1-benzofuran-3-one; C_21_H_22_O_11_, CAS 210050-28-5) from Molport (Riga, Latvia), and kaempferol-3-*O*-galactoside (C_21_H_20_O_11_, CAS 480-10-4) from Cymit Química (Barcelona, Spain). All compounds were delivered in powder form and then dissolved in 100% methanol before HPLC-MS/MS. **Supplementary Figure S3** shows the structures of all compounds quantified in this study.

#### 2.2.2 Plant material from greenhouse experiments

Potato plants (cv. ‘Quarta’ and ‘Record’) for secondary metabolite quantification, metabolomics and transcriptomics were grown in a greenhouse with a 16-h photoperiod (supplemented using lamps), and a daytime air temperature of 22–31 °C (**Supplementary Figure S4**). Plants were cultivated individually in 26-cm diameter pots containing substrate ED73 (Einheitserde Werkverband, Sinntal-Altengronau, Germany). Irrigation was applied via a drip pipe system with a daily volume of 30 mL. Flowers, small berries (< 1.5 cm diameter), and big berries (≥ 1.5 cm diameter) were collected in the morning (~9 am) from three independent plants and immediately frozen in liquid nitrogen. Before metabolite and RNA extraction, frozen plant material was ground to a fine powder using an MM 400 mill (Retsch, Haan, Germany) under liquid nitrogen and preserved at –80 °C for further analysis.

#### 2.2.3 Glycoalkaloid quantification

*Extraction*: Plant tissue (50 mg, ± 10%) was weighed into cold 2-mL microcentrifuge tubes and topped up with 1.5 mL 70% methanol. The tubes were incubated on an Eppendorf (Hamburg, Germany) thermomixer at 1400 rpm and 22 °C for 5 min before centrifugation (2,250 x g, 5 min, 4 °C). The supernatant was passed through a 0.2-µM syringe filter before LC-MS/MS analysis. Flower samples were diluted 1:5 using water:methanol (3:1).

*Quantification:* For quantification, we considered three biological replicates per group (flower, small berry and big berry), each separated in duplicate using an Agilent 1260 Infinity HPLC system (Agilent Technologies Germany, Waldbronn, Germany) equipped with a Nucleoshell RP 18 column (100 x 4.6 mm, 2.7 µm, Macherey-Nagel, Düren, Germany). The mobile phase was a mixture of (A) water and (B) ACN, both modified with 0.1% formic acid (FA). The gradient elution was 0–10 min, 10% B; 10–10.10 min, 50% B; 10.10–12.50 min, 90% B; 12.50–12.60 min, 90% B; 12.60–15 min, 10% B. The flow rate was 1 mL/min, the column temperature 25 °C, and the injection volume 10 µL. Detection was performed using the triple quadrupole mass spectrometer 6420 (Agilent, Santa Clara, CA, United States) equipped with an electrospray ionization (ESI) source in positive mode. The capillary voltage was set to 5.0 kV. The gas temperature was 300 °C. The gas flow was set to 10 L/h and the nebulizer pressure to 30 psi using nitrogen in both cases.

Compounds were detected by multiple reaction monitoring (MRM) in two-time segments: (1) start time at 0 min for glycosides (α-solanine, α-chaconine and α-solamargine); and (2) start time at 8 min for aglycones (solanidine and solasodine). The spray voltage was 5000 V, with a gas temperature of 300 °C. Gas flow was set to 10 L/min, and the nebulizer at 30 psi. Detailed MRM transitions, fragmentation voltages and collision energies are provided in **Supplementary Table S1**. Calibration curves were obtained by injecting known concentrations (2.5–50 nM; R^2^ ≥ 0.9971) of a reference mixture containing all five glycoalkaloids. Quantification was based on the final concentration determined by LC-MS/MS. Validation parameters of the LC-MS/MS method are shown in the **Supplementary Material** (**Supplementary Table S2**).

### 2.3 Untargeted analysis of secondary metabolites

Metabolomic analysis of potato flowers and berries from cultivars ‘Quarta’ and ‘Record’ was carried out by MetasysX (Potsdam, Germany), using a Waters (Milford, MA, USA) Acquity reversed-phase ultra-performance liquid chromatography (RP-UPLC) system equipped with C18 columns. This was coupled to an Exactive mass spectrometer (Thermo Fisher Scientific, Waltham, MA, USA) with an ESI source for positive and negative modes and an Orbitrap mass analyzer. Initial data processing was also carried out by MetasysX and was provided as tissue-median normalized peak intensities. Peaks were extracted and aligned according to retention time (RT) and mass-to-charge (m/z) ratio. Isotopic peaks, in-source fragments and redundant derivatives were filtered out. Data assignment (mass and RT tolerance: maximum of 3.45 ppm and 0.072 minutes of deviation, respectively) was achieved using an in-house database of MetasysX that considered the m/z and RT of 7500 reference compounds, as well as 1500 lipids and sugar esters putatively annotated (according to their “precursor m/z, fragmentation spectrum, and elution patterns”). Quality control steps involved filtering the data to remove isotopic peaks, in-source fragments of the analytes, and redundant peaks (less intense adduct of the same analyte). Data, given as peak intensities, were filtered down manually, removing unassigned compounds and keeping only those metabolites present in all three replicates of each group. Three secondary metabolites (coniferin, metabolite P and kaempferol-3-*O*-galactoside) were chosen for further quantification based on potential bioactivity and novel description in potato.

#### 2.3.1 Quantification of phenolic compounds

*Extraction*: Phenolic compounds were extracted according to Salem et al. (2016) with modifications. Plant tissue (100 mg, ± 10%) was weighed into cold 2-mL microfuge tubes and topped up with 1 mL of methyl *tert*-butyl ether (MTBE) + methanol (3:1). The tubes were vortexed for 1 min and incubated on a thermomixer at 300 rpm and 4 °C for 45 min before sonication (15 min). We added 650 µL of water + methanol (3:1), and vortexed for 1 min before centrifugation (22,300 x g, 5 min, 4 °C). We transferred 400 µL of the non-polar and polar phases to a new microcentrifuge tube, evaporated the solvent in a sample concentrator and re-dissolved the samples in water:methanol (3:1). The samples were then passed through a 0.2 µM syringe filter for LC-MS/MS analysis. Flower samples were diluted at a ratio of 1:2 in water:methanol (3:1).

*Quantification:* The experimental design for phenolic compounds followed that used for glycoalkaloids, with three biological replicates per group (flower, small berry and big berry) running in duplicate on a Waters Acquity UHPLC system coupled to a Waters Xevo TQ-S triple-quadrupole mass spectrometer (Waters Technologies Corp., MA, USA). The column and elution solvents were the same as for glycoalkaloids. The gradient program was as follows: 0–10 min, 90% - 50% A; 10 - 10,10 min, 10% A; 10,10 - 12,50 min, 10% A; 12,50 - 12,60 min, 90% A; 12,60–15 min, 90% A. The electrospray ionization (ESI) interface of the mass spectrometer was driven in the positive mode. The capillary voltage was set to 2.5 kV. The cone voltage was 25 V. The desolvation temperature and source temperature was 600 °C and 150 °C, respectively. The desolvation gas flow was set to 1000 L/h and the cone gas flow at 150 L/h using nitrogen in both cases. Mass spectrometric detection in the SIR/MRM mode was applied for quantification of the phenolic compounds (**Supplementary Table S3**). Argon was used as the collision gas at a flow of 0.15 mL/min. A calibration curve was prepared by injecting known concentrations (0.5–50 µM; R^2^ ≥ 0.9958) of a mixture containing all three phenolic compounds. Validation parameters of the LC-MS/MS method are shown in the **Supplementary Material** (**Supplementary Table S4**). Each sample was extracted and analyzed in duplicate. Extract concentrations were converted into mg/g fresh weight and averaged.

### 2.4 Differential gene expression analysis from potato flowers and berries

Total RNA was extracted from samples from cultivars ‘Quarta’ (Q) (flower (F), small berry (SB) and big berry (BB)) and ‘Record’ (R) (flower (F) and small berry (SB)) using the RNeasy Plant Mini Kit (Qiagen, Hilden, Germany), including the RNA-free DNase I step (Qiagen) to remove DNA, followed by RNA cleanup using the kit. Biological samples correspond to three plants per cultivar (Q, R) and group (F, SB, BB). Samples were also assessed for RNA integrity on an Agilent RNA 6000 Nano Bioanalyzer (Agilent Technologies, Santa Clara, CA, United States), and those with RNA integrity number (RIN) ≥ 9.1 were selected for RNA sequencing (RNA-Seq) by Illumina at Novogene (Munich, Germany). After sequencing, quality score Q30 ranged between 91.86% and 94.61% across all 15 samples and sequencing lanes. According to Novogene, Q30 is calculated by dividing the total number of bases with a Phred score greater than 20 or 30 by the total number of sequenced bases.

Adapters were trimmed using Trimmomatic v0.39 (Beier et al., 2026), and a second round of quality control with FastQ v0.12.0 (Wingett and Andrews, 2018) confirmed the quality improvement with consistently high per base sequence quality across the samples. Trimmed reads were mapped to the *Solanum tuberosum* reference genome SolTub_3.0 (Ensembl Plants) and quantified using Kallisto v0.50.1 (Bray et al., 2016). Differential gene expression was analyzed in R using DESeq2 from the Bioconductor Biobase suite using transcript-level abundance estimated by Kallisto. The following contrasts were considered: QF vs QSB, QF vs QBB, QSB vs QBB, RF vs RSB, QF vs RF, and QSB vs RSB. Genes with low expression (fewer than 10 read counts) were filtered out prior to analysis. The remaining data were normalized using a variance-stabilizing transformation (VST). Based on the genome annotation file SolTub_3.0.0.62.gff3 (Ensembl Plants), a transcript-to-gene mapping table was created using the R rtracklayer package and imported with tximport. Significantly differentially expressed genes (DEGs) were defined as those with an adjusted p-value < 0.01 and |log2-fold change| > 1. Lists of unique and commonly expressed genes between groups were generated using Venny v2.1 (Oliveros, 2007). Enrichment (one-sided Fisher’s exact test, over-representation, and false discovery rate (FDR) < 0.05) was determined using Mercator4 (Schwacke et al., 2019) based on the output of the protein annotation function of Mercator4 v7.

### 2.5 Statistical analysis

All data were analyzed in R v4.5.2 (R Core Team, 2026) and RStudio v2026.01.0 + 392. Data normality and homoscedasticity were pre-tested using the Shapiro-Wilk and Levene’s tests. *Field analysis:* by-product yield was analyzed using linear models with year and block as fixed effects. Tuber yield was analyzed using linear mixed-effects models with fixed effects being year, treatment and their interaction (year x treatment), and random effect being block nested within year. Estimated marginal means and Tukey test were used as *post-hoc* (p < 0.05). *Metabolite quantification:* field samples were analyzed with linear mixed-effects models with year, tissue, and their interaction (year x tissue) as fixed effects, with block nested within year as a random effect and estimated marginal means and Tukey test as *post-hoc* (p < 0.05). For field samples, each treatment within each block contained one biological sample composed of a pool of 10 plants. Greenhouse samples were analyzed with one-way ANOVA followed by Tukey’s *post-hoc* test (p < 0.05) for Quarta, and t-test (p < 0.05) for Record. Biological replicates in greenhouse samples represent three independent plants. *Metabolomics:* to test the effect of tissue, cultivar and their interaction on different key secondary metabolites, the raw data obtained from MetasysX with and without unassigned peaks served as input for the metadata of MetaboAnalyst v6.0 (Pang et al., 2024). The data were filtered for low quality (removal of features with > 20% missing values), low repeatability (relative standard deviation > 20%), low variance (interquartile range with 20% filtered out) and low abundance (based on mean intensity value). We then applied left-censored data estimation, where the limit of detection replaced the missing values with 1/5 of the minimum positive values of individual features. Peak intensities from raw data were sample-normalized by median and log_2_-transformed to reduce heteroscedasticity. A two-way ANOVA was applied for tissue and cultivar, and p-values were adjusted with an FDR (Benjamini-Hochberg < 0.05). *Transcriptomics:* data were analyzed as described above using the Bioconductor packages DESeq2 (Love et al., 2014), tximport (Soneson et al., 2016), and rtracklayer (Lawrence et al., 2013).

## 3 Results

### 3.1 Feasibility of potato by-product harvesting and glycoalkaloid quantification

We conducted two field trials using potato cultivars ‘Quarta’ and ‘Granola’ in two consecutive years (2022 and 2023) to evaluate the generation of by-products and the impact of by-product harvest on tuber yield as well as the extractability of glycoalkaloids from field samples. Starting with by-product production (fresh material), as expected due to the heavier weight, the production of berries considerably surpassed the production of flowers in both years. In 2022, both cultivars yielded similar amounts of flowers (‘Quarta’ 17.09 kg/ha; ‘Granola’ 12.86 kg/ha) and berries (‘Quarta’ 376.68 kg/ha; ‘Granola’ 354.31 kg/ha). However, in 2023, ‘Quarta’ substantially increased its production of flowers (77.38 kg/ha) whereas the production of berries was reduced by more than 50% (171.84 kg/ha). ‘Granola’ maintained flower yields (12.87 kg/ha) but reduced the production of berries to 260.40 kg/ha (**Figure 1**).

**Figure 1:**
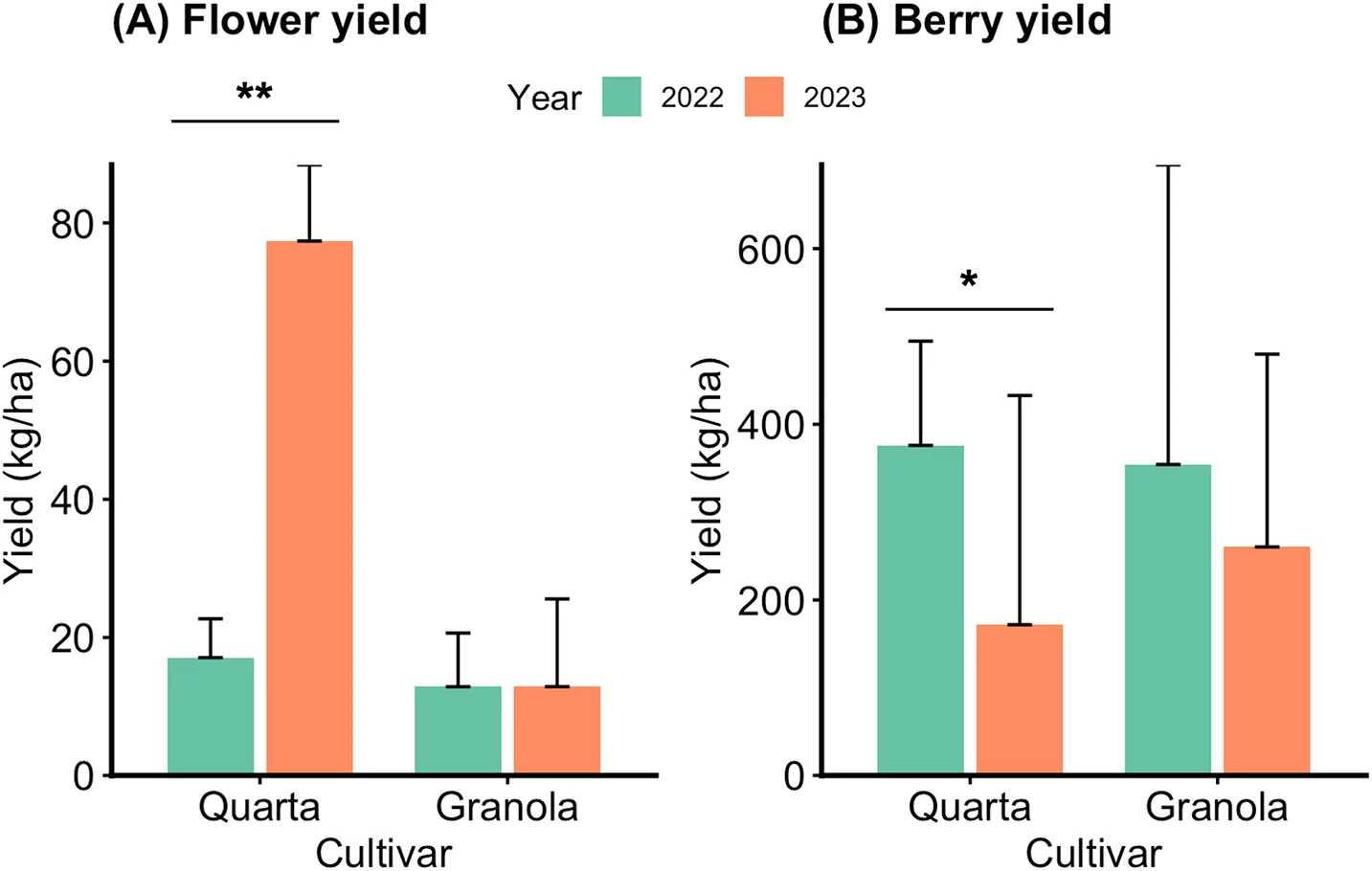
Flower **(A)** and berry **(B)** yield (kg/ha) in field trials conducted in 2022 and 2023 with cultivars ‘Quarta’ and ‘Granola’. Asterisks indicate significant differences between years for the same cultivar and tissue as determined by linear-mixed effects models followed by Tukey’s test (*p < 0.05, **p < 0.01). Data are means ± SD for by-product yield per block with n = 4 blocks (2022) and n = 6 blocks (2023).

**Figure 2** shows the impact of harvesting flowers and berries before tuber harvest on the overall tuber yield. Where no by-product was harvested, the tuber yield was 15,785.71 and 35,071.21 kg/ha for ‘Quarta’, and 22512.99 and 45648.18 kg/ha for ‘Granola’ in 2022 and 2023, respectively. In most cases, tuber yield remained at the same levels with or without the harvest of by-products before the tuber harvest. However, in 2023, the harvesting of ‘Quarta’ berries in advance significantly reduced the tuber yield to 28,876.75 kg/ha.

**Figure 2:**
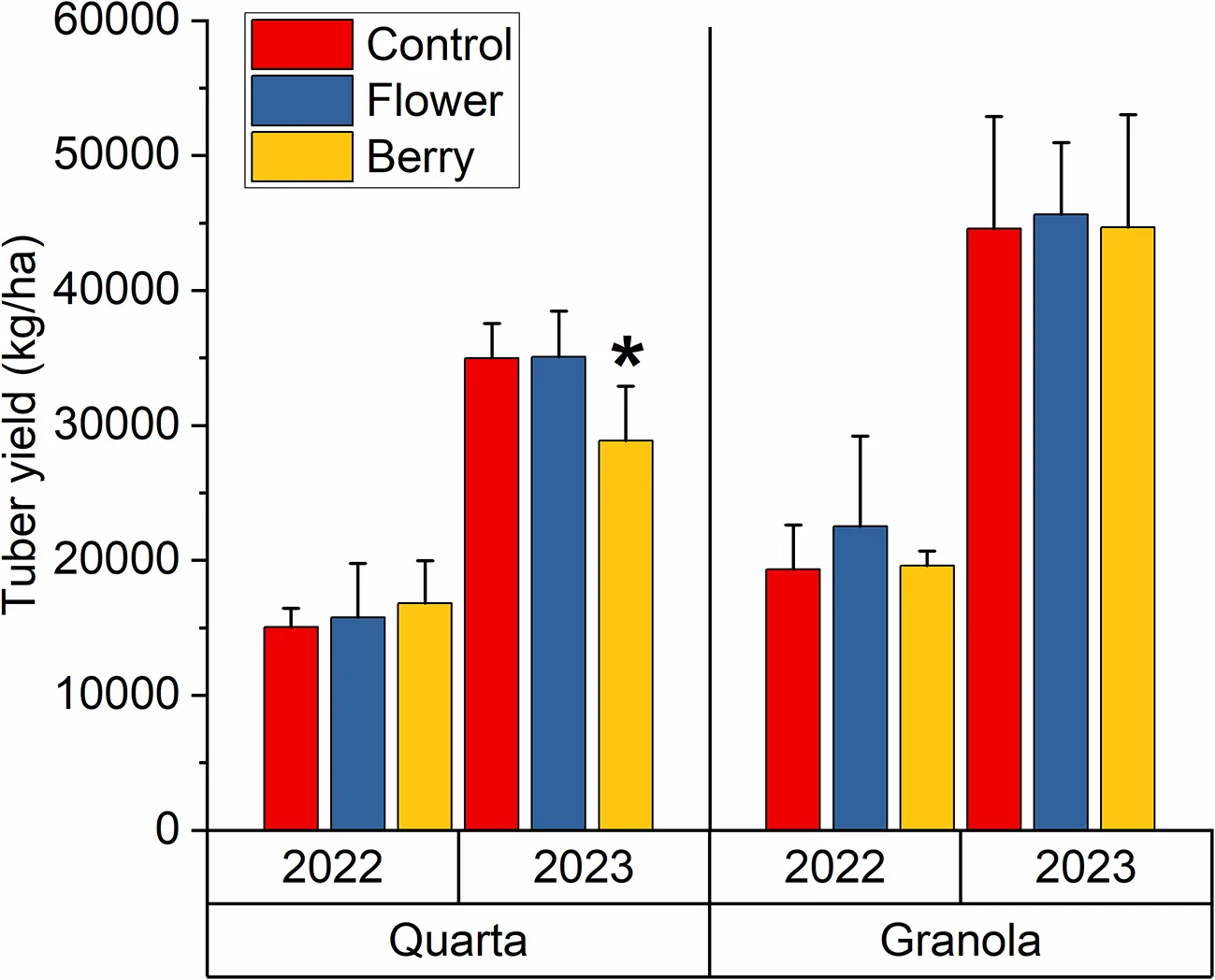
Impact of early harvest of by-products on tuber yield (kg/ha) in two field trials (2022 and 2023). Asterisk (*) indicates differences between treatments for the same year and cultivar as determined by linear-mixed effects models followed by Tukey’s test (p < 0.05). Data are means ± SD for total tuber yield per block with n = 4 blocks (2022) and n = 6 blocks (2023).

To evaluate the quantity of glycoalkaloids in field samples, flowers and berries from both field trials (2022 and 2023) were analyzed by HPLC-MS to detect five target compounds (**Table 1**). We did not detect the spirosolane aglycone solasodine or its glycoalkaloid counterpart α-solamargine, or the solanidane aglycone solanidine, in either flowers or berries. In contrast, α-solanine and α-chaconine were detected in both tissues, with strong content differences. Potato flowers contained 4–7× more α-solanine and 3–10× more α-chaconine than potato berries, depending on the cultivar.

**Table 1:** Glycoalkaloids quantified by HPLC-MS/MS in potato by-products from two field trials (2022 and 2023).

Year	Cultivar	Tissue		Glycoalkaloids (mg/100 g FW)		
			α-solanine	α-chaconine	TGC	Ratio C:S
2022	‘Quarta’	Flower	232.74 ± 36.95 aB	383.43 ± 47.01 aA	616.17	1.65 ± 0.06 a
		Berry	35.30 ± 19.31 bB	40.12 ± 22.11 bA	75.42	1.14 ± 0.04 b
	‘Granola’	Flower	160.21 ± 19.89 aB	353.08 ± 36.74 aA	513.29	2.21± 0.08 a
		Berry	30.03 ± 3.17 bB	45.61± 4.42 bA	75.64	1.52 ± 0.09 b
2023	‘Quarta’	Flower	66.90 ± 9.65 aB	91.59 ± 13.27 aA	158.49	1.37 ± 0.03 a
		Berry	9.92 ± 1.47 bB	13.97 ± 3.40 bA	23.89	1.39 ± 0.14 a
	‘Granola’	Flower	41.51 ± 7.95 aB	38.76 ± 8.43 aA	80.27	0.93 ± 0.04 a
		Berry	9.59 ± 1.97 bB	11.22 ± 2.99 bA	20.81	1.16 ± 0.08 b

Different lowercase letters indicate significant differences between tissues for a given compound within the same cultivar and year. Different uppercase letters indicate significant differences between the concentrations of α-solanine and α-chaconine within the same tissue, cultivar, and year. Data were analyzed using linear-mixed effects models followed by Tukey’s *post hoc* (p < 0.05). TGC = total glycoalkaloid content. Data are means ± SD for n = 4 (2022) and n = 6 (2023).

Considering the total glycoalkaloid content (TGC) as the sum of α-solanine and α‐chaconine, the highest TGC was recorded in ‘Quarta’ flowers collected in 2022 (616.17 mg/100 g FW) and the lowest in ‘Granola’ berries collected in 2023 (20.81 mg/100 g FW). The TGC varied strongly between years, with values in all tissues 68% lower in 2023 than 2022. The relative content of glycoalkaloids also varied within the same tissue. We found that α-chaconine was generally more abundant than α-solanine, with ratios varying between 0.93 and 2.21 in flowers, and between 1.14 and 1.52 in berries.

### 3.2 Plant development and tissue impact on glycoalkaloid content

Given the differences in glycoalkaloid content in flowers and berries from field trials, we investigated whether plant development also affected the content of plants grown under controlled conditions in the greenhouse. In this case, glycoalkaloids were extracted from flowers, small berries (< 1.5 cm diameter) from cultivars ‘Quarta’ and ‘Record’, and big berries (≥ 1.5 cm diameter) from cultivar ‘Quarta’ and quantified by HPLC-MS/MS (**Table 2**). The cultivar ‘Record’ did not produce big berries in the greenhouse. Therefore, the comparisons for this cultivar did not extend beyond the small berry stage. The cultivar ‘Granola’ was not included in the quantification analysis from greenhouse samples due to the lack of berries (small or big) in this cultivation site.

**Table 2:** HPLC-MS/MS analysis of glycoalkaloids in potato tissues from plants cultivated in the greenhouse.

Cultivar	Tissue		Glycoalkaloids (mg/100 g FW)		
		α-solanine	α-chaconine	TGC	Ratio C:S
‘Quarta’	Flower	136.87 ± 22.20 aB	257.08 ± 31.63 aA	393.95	1.89 ± 0.08 a
	Small berry	29.71 ± 9.98 bA	42.02 ± 10.94 bA	71.73	1.44 ± 0.15 b
	Big berry	32.02 ± 9.16 bA	38.08 ± 9.54 bA	70.10	1.20 ± 0.04 b
‘Record’	Flower	112.41 ± 12.26 aB	373.39 ± 29.51 aA	485.80	3.33 ± 0.11 a
	Small berry	50.58 ± 8.11 bB	111.15 ± 16.35 bA	161.73	2.20 ± 0.03 b

Different lowercase letters indicate significant differences between tissues for a given compound within the same cultivar and year. Different uppercase letters indicate significant differences between the concentrations of α-solanine and α-chaconine within the same tissue, cultivar and year. TGC = total glycoalkaloid content. Small berry: Ø < 1.5 cm. Big berry: Ø ≥ 1.5 cm. Statistical significance was determined by one-way ANOVA with Tukey’s *post hoc* test (p < 0.05) (‘Quarta’) and t-test (p < 0.05) (‘Record’). Data are means ± SD for n = 3.

Plants cultivated under controlled conditions behaved similarly to those in the field, with flowers accumulating higher glycoalkaloid concentrations than berries. For cultivar ‘Quarta’, flowers contained almost 5× more α-solanine and 7× more α-chaconine than small and big berries, respectively, whereas the content of α-solanine and α-chaconine in ‘Record’ flowers was 2× and 4× higher than in small berries, respectively.

In ‘Quarta’, we observed a TGC of 393.95 mg/100 g FW in flowers, 71.73 mg/100 g FW in small berries, and 70.10 mg/100 g FW in big berries. In ‘Record’, the TGC was 485.8 mg/100 g FW in flowers and 161.73 mg/100 g FW in small berries. The α-chaconine content was also higher than that of α-solanine, especially in flowers and small berries (‘Record’). Ratios varied between 1.89 and 3.33 in flowers, and 1.44 and 2.20 in small berries. No differences were recorded between small and big berries in ‘Quarta’.

### 3.3 Identification of other metabolites in potato by-products

Untargeted metabolomic profiling (RP-UPLC-MS/MS) in greenhouse samples from cultivars ‘Quarta’ and ‘Record’ was carried out to evaluate how potato by-products might also be a source of other added-value compounds. MetasysX detected 3634 peaks (1875 in positive mode and 1759 in negative mode), 516 of which were assigned to known metabolites, including four putatively annotated metabolites (as previously mentioned in section 2.3). Among these identified features, 92% (476) presented a strong signal in all three replicates per group, and these were considered for further analysis. Flower samples from both cultivars featured more identified metabolites (404) than small berries (391) and big berries (289). Overall, the ‘Record’ samples contained more identified metabolites than the ‘Quarta’ samples. The complete list of 3634 peaks is provided in **Supplementary Table S5**.

We used tissue-median normalized peak intensities for principal component analysis (PCA) to reveal differences among samples, with PC1 explaining most (89.5%) of the variance and PC2 explaining 5.1% (**Figure 3**). Samples formed clusters reflecting their developmental stage/organ type and were also related to the cultivar. For example, we observed good separation between the flower and berry samples but the ‘Quarta’ and ‘Record’ flowers overlapped in a single cluster, showing their similarity. The ‘Quarta’ and ‘Record’ small berries formed separate clusters, as did the ‘Quarta’ big berries, indicating developmental and cultivar-specific differences. ‘Record’ small berries showed the largest variability among replicates.

**Figure 3:**
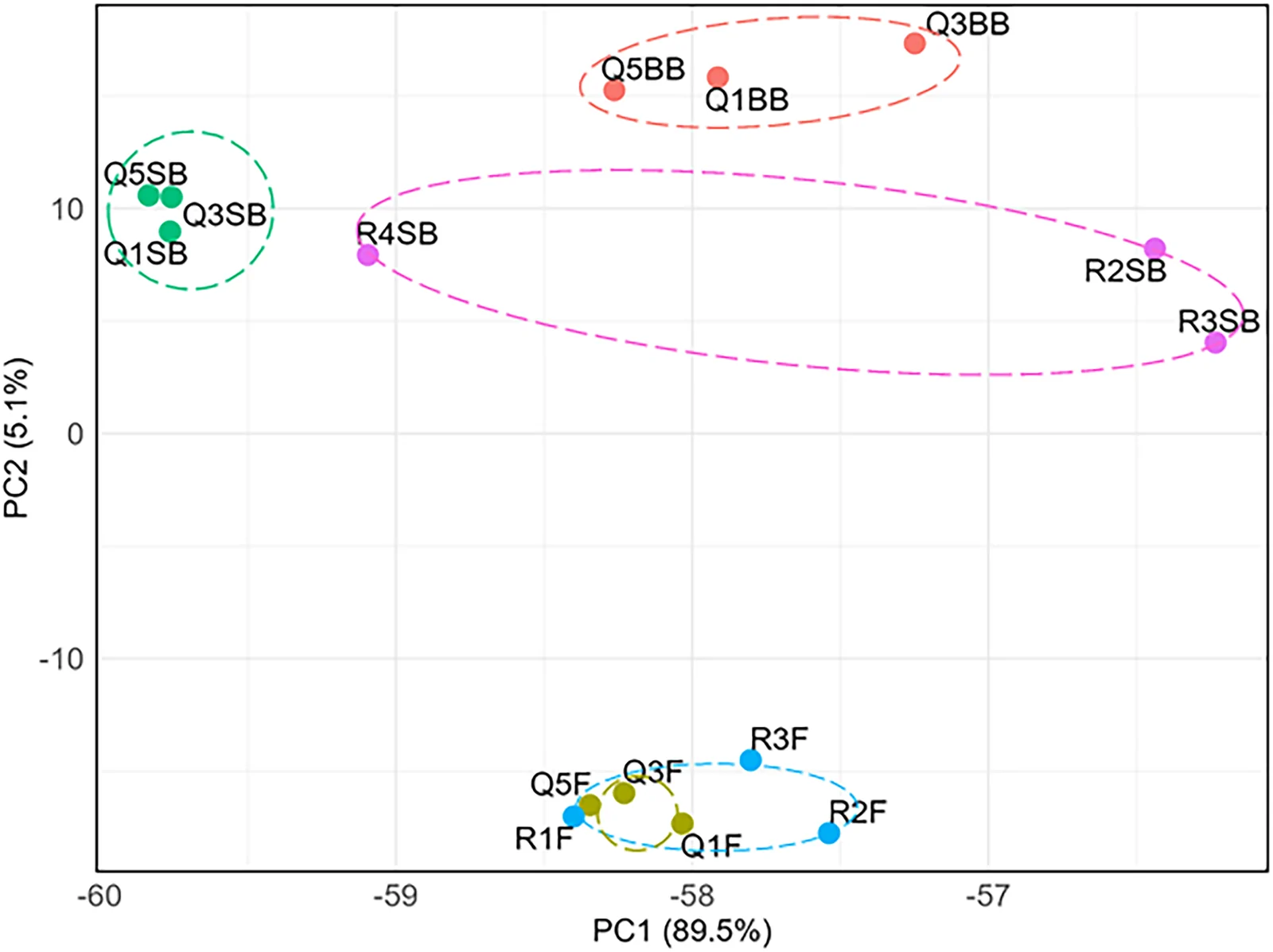
Principal component analysis (PCA) of metabolomics data with tissue-median normalized intensities. Q, ‘Quarta’; R, ‘Record’; F, flower; SB, small berry; BB, big berry. Color of dots and dashed circles = olive green, QF; teal, QSB; salmon, QBB; bright blue, RF; magenta, RSB.

#### 3.3.1 Key metabolites across tissues and cultivars

The average peak intensity of our samples ranged from 4.14×10^4^ to 6.89×10^9^. **Table 3** lists the top metabolites by peak intensity in each cultivar and tissue (QF, QSB, QBB, RF and RSB). Metabolites A–Y refer to compounds with long names (listed in **Supplementary Table S6**), mainly belonging to flavonoids, organooxygen compounds, and carboxylic acid derivatives classes. MetasysX detected a mixture of primary and secondary metabolites whose peak intensities increased or decreased during development. The upper part of **Table 3** shows metabolites present at higher intensity among most samples, representing amino acids, nucleic acids, alkaloids, and phenolic compounds. The lower part of the table shows metabolites with lower peak intensities (1.0×10^7^) that could be detected only in one tissue or in one cultivar.

**Table 3:** Top metabolites present in potato by-products by average peak intensity (n = 3).

Metabolite	QF	QSB	QBB	RF	RSB
Putative solanine	3.80×10^9^	6.57×10^9^	6.89×10^9^	2.08×10^8^	1.65×10^9^
*N*-Methylnicotinic acid	3.42×10^9^	2.96×10^9^	4.15×10^9^	5.51×10^9^	3.61×10^9^
l-Proline	3.36×10^9^	2.60×10^8^	2.91×10^8^	2.97×10^9^	1.17×10^8^
Quinic acid	1.97×10^9^	2.32×10^9^	3.07×10^9^	2.25×10^9^	1.88×10^9^
l-Malic acid	1.84×10^9^	1.14×10^9^	2.50×10^9^	1.70×10^9^	7.16×10^8^
(*)l-Gluatamine/l‐alanylglycine	1.79×10^9^	9.56×10^8^	2.81×10^9^	1.25×10^9^	3.50×10^8^
Choline	1.69×10^9^	5.23×10^8^	1.23×10^9^	1.26×10^9^	8.74×10^8^
(*) Sucrose/Turanose	7.18×10^8^	5.10×10^8^	9.71×10^8^	5.90×10^8^	3.52×10^8^
Adenine	6.53×10^8^	9.15×10^7^	1.11×10^8^	3.80×10^8^	3.27×10^7^
Putative solanandaine	5.74×10^8^	1.63×10^8^	5.52×10^7^	3.29×10^8^	4.70×10^7^
(*) d-Gluconic acid	5.66×10^8^	4.37×10^8^	5.09×10^8^	3.39×10^8^	2.15×10^8^
Metabolite B	4.71×10^8^	2.06×10^8^	3.14×10^7^	6.69×10^8^	5.10×10^8^
l-Glutamic acid	4.17×10^8^	2.24×10^8^	2.84×10^8^	3.82×10^8^	1.70×10^8^
(*) Rutin	4.13×10^8^	7.55×10^7^	3.90×10^6^	4.58×10^8^	1.80×10^8^
Betaine	3.32×10^8^	3.35×10^8^	4.71×10^8^	6.46×10^8^	3.44×10^8^
Metabolite D	3.12×10^8^	2.10×10^8^	3.78×10^7^	4.06×10^8^	4.21×10^8^
Citric acid	2.95×10^8^	5.63×10^8^	8.96×10^8^	1.94×10^8^	1.54×10^8^
Chlorogenic acid	2.70×10^8^	7.18×10^8^	5.29×10^7^	3.78×10^8^	5.77×10^8^
Metabolite F	2.54×10^8^	1.58×10^7^	8.32×10^6^	3.78×10^8^	7.66×10^7^
d-Saccharic acid	2.29×10^8^	8.05×10^7^	9.92×10^7^	8.96×10^7^	3.90×10^7^
l-Tyrosine	2.03×10^8^	7.95×10^8^	1.02×10^9^	1.13×10^8^	1.29×10^8^
Metabolite A	1.98×10^8^	6.46×10^8^	4.31×10^7^	3.51×10^8^	6.27×10^8^
l-Isoleucine	1.89×10^8^	6.16×10^8^	1.17×10^9^	1.40×10^8^	2.29×10^8^
(*) l-Asparagine	1.87×10^8^	6.27×10^7^	1.25×10^8^	1.51×10^8^	1.39×10^8^
(*) l-Valine	1.79×10^8^	4.99×10^8^	1.04×10^9^	2.43×10^8^	2.87×10^8^
Metabolite C	1.76×10^8^	2.72×10^7^	1.27×10^7^	6.43×10^8^	2.42×10^7^
l-Tryptophan	1.72×10^8^	7.72×10^8^	9.89×10^8^	1.08×10^8^	1.71×10^8^
Leucine	1.70×10^8^	3.03×10^8^	5.69×10^8^	1.32×10^8^	1.39×10^8^
Metabolite K	1.50×10^8^	6.66×10^7^	1.24×10^7^	2.21×10^8^	1.30×10^8^
Metabolite L	1.27×10^8^	5.08×10^5^	1.69×10^5^	1.38×10^7^	2.27×10^5^
Metabolite I	1.16×10^8^	6.63×10^7^	1.05×10^8^	1.07×10^8^	3.38×10^7^
Metabolite E	1.04×10^8^	1.74×10^7^	1.06×10^7^	3.95×10^8^	2.03×10^8^
*N*,*N*-Dimethylglycine	6.23×10^7^	3.84×10^8^	1.08×10^9^	8.15×10^7^	8.50×10^7^
l-Methionine	7.32×10^7^	2.72×10^8^	9.14×10^8^	1.13×10^7^	8.16×10^7^
l-Arginine	6.07×10^7^	2.60×10^8^	9.49×10^8^	5.27×10^7^	6.31×10^7^
Metabolite G	2.72×10^7^	1.65×10^8^	1.67×10^8^	3.19×10^7^	1.15×10^8^
(*) d-Galacturonic acid	3.78×10^7^	1.38×10^8^	8.84×10^7^	5.02×10^7^	4.88×10^7^
Histamine	4.39×10^7^	1.13×10^8^	6.75×10^7^	5.70×10^7^	1.23×10^8^
putative Dehydrosolanine	6.91×10^7^	1.13×10^8^	5.49×10^7^	1.72×10^8^	1.21×10^8^
l-Lysine	1.71×10^7^	6.16×10^7^	1.69×10^8^	1.06×10^7^	2.32×10^7^
Pyroglutamic acid	1.82×10^7^	2.11×10^7^	1.45×10^8^	1.18×10^7^	9.74×10^6^
(*) Myo-inositol/d-tagatose	8.18×10^7^	6.76×10^7^	1.39×10^8^	4.34×10^7^	1.32×10^7^
Metabolite H	1.91×10^7^	7.29×10^7^	1.29×10^8^	1.13×10^7^	1.53×10^7^
(*) l-Threonine	3.40×10^7^	3.54×10^7^	1.01×10^8^	2.66×10^7^	1.23×10^7^
Neochlorogenic acid	1.17×10^7^	5.33×10^7^	2.35×10^6^	4.60×10^7^	1.17×10^8^
Metabolite M	9.65×10^7^	2.46×10^6^	1.18×10^6^	8.38×10^7^	4.76×10^5^
Metabolite U	8.32×10^7^	6.42×10^7^	8.67×10^7^	8.02×10^7^	1.35×10^7^
Metabolite J	7.26×10^7^	2.96×10^7^	4.52×10^7^	7.76×10^7^	3.56×10^7^
l-Histidine	7.21×10^7^	3.37×10^7^	9.84×10^7^	3.44×10^7^	2.17×10^7^
Guanosine	6.32×10^7^	3.38×10^6^	3.68×10^7^	4.80×10^7^	1.61×10^7^
Metabolite O	5.63×10^7^	2.45×10^7^	4.81×10^6^	7.98×10^7^	3.69×10^7^
d-Fucose	5.58×10^7^	1.10×10^7^	4.53×10^7^	4.69×10^7^	3.96×10^6^
Metabolite V	5.23×10^7^	4.68×10^7^	–	3.20×10^7^	–
*N*-Acetylputrescine	5.18×10^7^	8.54×10^6^	9.92×10^6^	5.44×10^7^	8.67×10^6^
*N*-(2-Hydroxyethyl)-β-alanine	5.16×10^7^	2.34×10^7^	3.05×10^7^	2.50×10^7^	1.15×10^7^
Metabolite P	5.00×10^7^	1.73×10^5^	–	1.93×10^7^	–
Metabolite X	4.58×10^7^	6.68×10^5^	–	2.49×10^7^	4.29×10^5^
(*) d-Fructose-6-phosphate	1.82×10^7^	5.27×10^7^	7.29×10^7^	1.79×10^7^	1.44×10^7^
(*) l-Alanyl-l-alanine	2.22×10^7^	3.58×10^7^	7.38×10^7^	8.72×10^6^	7.59×10^6^
Metabolite N	2.36×10^7^	3.46×10^7^	2.01×10^7^	4.36×10^7^	7.12×10^7^
Spermidine	2.44×10^7^	3.25×10^7^	2.35×10^7^	4.08×10^7^	1.93×10^7^
Allantoic acid	1.78×10^7^	2.79×10^7^	2.42×10^7^	2.64×10^7^	1.26×10^7^
(*) *N*-Methyl-l-glutamic acid	1.24×10^7^	2.75×10^7^	3.01×10^7^	3.81×10^6^	1.27×10^7^
Acetylcholine	5.42×10^6^	4.09×10^6^	7.00×10^7^	5.78×10^6^	4.49×10^6^
l-Glutamyl-l-glutamine	1.42×10^7^	1.31×10^7^	5.26×10^7^	9.15×10^6^	2.32×10^6^
Adenosine monophosphate	8.76×10^5^	2.54×10^7^	4.25×10^7^	1.70×10^7^	1.34×10^7^
(*) l-Threonyl-l-alanine	2.81×10^7^	6.32×10^6^	3.76×10^7^	2.67×10^7^	3.71×10^6^
Uridine 5′-monophosphate	2.14×10^6^	6.32×10^6^	3.70×10^7^	1.31×10^7^	6.26×10^6^
Metabolite Q	3.95×10^7^	1.32×10^7^	1.73×10^7^	9.02×10^7^	2.65×10^7^
Metabolite T	3.37×10^7^	1.65×10^7^	1.54×10^7^	4.93×10^7^	2.26×10^7^
l-Valyl-l-asparagine	2.32×10^7^	5.65×10^6^	6.67×10^6^	4.15×10^7^	6.55×10^6^
Metabolite R	2.85×10^5^	3.01×10^6^	–	3.13×10^7^	5.70×10^7^
Metabolite S	1.43×10^7^	1.56×10^7^	2.93×10^6^	2.90×10^7^	3.40×10^7^
Metabolite Y	5.61×10^6^	2.16×10^6^	3.77×10^6^	2.11×10^7^	1.89×10^7^

Colors indicate the peak intensity based on exponent values: red to dark green ≡ lower intensity (1.0×10^5^) to higher intensity (1.0×10^9^). (*) = Metabolites with 2–7 annotations (the other annotations are shown in **Supplementary Table S5**). (–) = Metabolites not assigned in specific tissue. Q, ‘Quarta’; R, ‘Record’; F, flower; SB, small berry; BB, big berry. Full names of metabolites A–Y are listed in **Supplementary Table S6**.

Glycoalkaloids were identified as the following “putative” compounds: putative solanine, putative solanandaine, putative dehydrosolanine, and putative solanine with hexose. In ‘Quarta’ samples, putative solanine was the alkaloid present at the highest intensity, with levels increasing from 3.80×10^9^ to 6.89×10^9^ during the progression from flower to big berry. For this cultivar, the other three alkaloids were less abundant, with peak intensities of 9.01×10^5^ to 5.74×10^8^. Putative solanine with hexose did not show up in the top metabolites but was present in all stages of ‘Quarta’ decreasing with progression from flower to big berry (QF 7.02×10^6^; QSB 2.57×10^6^; QBB 9.01×10^5^). In ‘Record’ samples, putative solanine was detected at lower levels than in ‘Quarta’, between 2.08×10^8^ and 1.65×10^9^, and putative solanine with hexose was detected only in small berries (4.97×10^6^), with similar intensity as ‘Quarta’ small berries (2.57×10^6^). Both cultivars contained putative solanandaine and dehydrosolanine, but only the first showed a decrease in intensity during development. Although a major glycoalkaloid found in potato tissues, α-chaconine was not annotated in the dataset, probably reflecting the lack of a spectral library reference rather than its absence in our samples. The alkaloid *N*-methylnicotinic acid also showed high peak intensities in all tissues of both cultivars, varying from 2.96×10^9^ to 5.51×10^9^ with high peak intensities in flowers and big berries. Other secondary metabolites ranked among the top 50 were phenolic compounds such as chlorogenic acid, neochlorogenic acid and rutin.

#### 3.3.2 Tissue and cultivar-specific metabolites

Statistical analysis of the untargeted metabolomics data showed that some metabolites accumulate differently throughout the plant. **Table 4** lists 18 secondary metabolites, most also included in the previous top metabolites list (**Table 3**), with the addition of kaempferol-3-*O*-galactoside due to its selection for quantification. Among these secondary metabolites, 50% can be considered “tissue-specific”. Their abundance (normalized means log_2_-transformed) match the data in **Table 3**, showing compounds like putative solanandaine, metabolites O (4,8-dihydroxy-1-naphthyl β-d-glucopyranoside), P (2,6-dihydroxy-2-[(4-hydroxyphenyl)methyl]-4-[(2S,3R,4S,5S,6R)-3,4,5-trihydroxy-6-(hydroxymethyl)oxan-2-yl]oxy-1-benzofuran-3-one), X (2-(3,4-dihydroxyphenyl)-3-[(2S,3R,4S,5S,6R)-4,5-dihydroxy-3-[(2R,3R,4R,5R,6S)-3,4,5-trihydroxy-6-methyloxan-2-yl]oxy-6-[[(2R,3R,4R,5R,6S)-3,4,5-trihydroxy-6-methyloxan-2-yl]oxymethyl]oxan-2-yl]oxy-5,7-dihydroxychromen-4-one) and K ((1S,3R,4S,5R)-1,3,5-trihydroxy-4-{[(2E)-3-(4-hydroxy-3-methoxyphenyl)-2-propenoyl]oxy}cyclohexanecarboxylic acid) decreasing as development progresses from flower to small berry and big berry. Others, such as neochlorogenic acid, metabolites D (3-[3,4-dihydroxy-6-(hydroxymethyl)-5-[3,4,5-trihydroxy-6-(hydroxymethyl)oxan-2-yl]oxyoxan-2-yl]oxy-2-(3,4-dihydroxyphenyl)-5,7-dihydroxychromen-4-one), and A ((1r,3R,4s,5S)-4-{[(2E)-3-(3,4-dihydroxyphenyl)-2-propenoyl]oxy}-1,3,5-trihydroxycyclohexanecarboxylic acid) peaked in abundance in the small berry, decreasing in big berries. Metabolite Y (methyl 2-[(2S,3R,4S,5S,6R)-3,4,5-trihydroxy-6-[[(2S,3R,4S,5R)-3,4,5-trihydroxyoxan-2-yl]oxymethyl]oxan-2-yl]oxybenzoate) was the only compound showing significance in the cultivar variable and in the interaction tissue × cultivar. The ‘Record’ cultivar accumulated more metabolite Y than ‘Quarta’.

**Table 4:** Evaluation of the different intensities of key secondary metabolites across tissues, cultivars and their interactions.

Class	Metabolite	Mean ± SD					ANOVA two-way (p.adj)		
		QF	QSB	QBB	RF	RSB	T	C	Int.
Steroid alkaloids	Putative Solanandaine	8.96 ± 0.15	7.70 ± 0.48	5.86 ± 1.62	8.03 ± 0.33	6.05 ± 0.97	4×10^-3^*	7×10^-2^	7×10^-1^
Caffeate derivatives	Neochlorogenic acid	2.80 ± 1.84	6.01 ± 0.74	0.90 ± 1.68	2.90 ± 4.25	7.36 ± 1.08	2×10^-2^*	7×10^-1^	8×10^-1^
Glycoside	Metabolite O	5.61 ± 0.22	5.01 ± 0.25	2.75 ± 0.03	5.86 ± 0.75	5.80 ± 0.78	3×10^-4^*	2×10^-1^	6×10^-1^
Aurone flavonoids	Metabolite P	5.45 ± 0.08	–2.15 ± 0.58	–3.89 ± 1.25	3.93 ± 0.31	–3.15 ± 2.05	4×10^-6^*	1×10^-1^	8×10^-1^
Flavonols	Rutin	8.19 ± 1.36	6.47 ± 0.85	1.48 ± 1.91	7.78 ± 2.16	8.08 ± 0.81	2×10^-3^*	6×10^-1^	5×10^-1^
Flavonols	Metabolite D	7.88 ± 1.12	8.08 ± 0.47	4.30 ± 2.45	7.79 ± 1.81	9.43 ± 0.30	2×10^-2^*	6×10^-1^	6×10^-1^
Flavonols	Metabolite X	5.31 ± 0.22	–0.17 ± 0.33	–2.73 ± 1.78	4.31 ± 0.29	–0.54 ± 0.50	4×10^-6^*	3×10^-1^	7×10^-1^
Organooxygen compounds	Metabolite A	6.96 ± 1.73	9.70 ± 0.45	5.24 ± 1.50	6.46 ± 3.68	10.00 ± 0.32	2×10^-2^*	9×10^-1^	8×10^-1^
–	Metabolite K	7.03 ± 0.18	6.45 ± 0.30	4.10 ± 0.16	7.35 ± 0.69	7.59 ± 0.85	3×10^-4^*	7×10^-2^	4×10^-1^
Cinnamic acid derivatives	Metabolite C	7.23 ± 0.33	5.13 ± 0.44	4.15 ± 0.33	8.97 ± 0.46	5.26 ± 0.68	7×10^-6^*	2×10^-2^*	6×10^-2^
Cinnamic acid derivatives	Metabolite S	3.61 ± 0.36	4.36 ± 0.27	2.01 ± 0.22	4.29 ± 0.99	5.70 ± 0.71	1×10^-3^*	3×10^-2^*	6×10^-1^
O-acyl-carbohydrate	Metabolite R	5.10 ± 0.07	4.09 ± 0.39	4.51 ± 0.61	6.17 ± 0.24	5.41 ± 0.48	2×10^-2^*	2×10^-3^*	7×10^-1^
Glycoside	Metabolite Y	2.28 ± 0.30	1.37 ± 0.74	2.37 ± 0.57	4.08 ± 0.17	4.98 ± 0.16	3×10^-1^	2×10^-5^*	3×10^-2^*
Naringenin derivatives	Metabolite M	6.38 ± 0.17	1.70 ± 0.33	0.60 ± 0.54	6.04 ± 0.35	–0.41 ± 0.52	3×10^-8^*	2×10^-3^*	3×10^-2^*
Flavonols	Kaempferol-3-*O*-galactoside	4.23 ± 0.42	0.57 ± 0.17	–0.49 ± 0.40	4.70 ± 0.31	3.13 ± 0.44	8×10^-7^*	2×10^-4^*	5×10^-3^*
Flavonols	Metabolite E	6.47 ± 0.45	4.54 ± 0.13	3.86 ± 0.24	8.31 ± 0.14	8.36 ± 0.43	5×10^-5^*	5×10^-7^*	4×10^-3^*
Flavonols	Metabolite F	7.76 ± 0.46	4.39 ± 0.21	3.50 ± 0.37	8.24 ± 0.24	6.92 ± 0.56	4×10^-6^*	4×10^-4^*	9×10^-3^*
–	Metabolite L	6.79 ± 0.31	–0.60 ± 0.46	–2.12 ± 0.31	3.38 ± 0.67	–1.56 ± 0.82	5×10^-8^*	3×10^-4^*	2×10^-2^*

Data are sample-median-normalized log_2_-transformed peak intensities and are displayed as means ± SD for n = 3. Data were analyzed by two-way ANOVA followed by FDR (Benjamini-Hochberg) correction (0.05), and asterisks (*) indicate significant differences regarding tissue, cultivar, or the interaction between these two variables. Q, ‘Quarta’; R, ‘Record’; F, flower; SB, small berry; BB, big berry; T, tissue; C, cultivar; Int., interaction tissue × cultivar. Names from metabolites A–Y can be found in **Supplementary Table S6**. Classes not present in the PubChem, KEGG, ChEBI, MolGenie, LOTUS or LIPID MAPS databases are shown with the symbol (–).

Given that both tissue and cultivar were significant for metabolites C ((3R,5R)-1,3,5-trihydroxy-4-[(E)-3-(4-hydroxyphenyl)prop-2-enoyl]oxycyclohexane-1-carboxylic acid), S (methyl (1S,3R,4R,5R)-3-[(E)-3-(3,4-dihydroxyphenyl)prop-2-enoyl]oxy-1,4,5-trihydroxycyclohexane-1-carboxylate) and R ((1S,3R,4R,5R)-3,4-bis[[(E)-3-(3,4-dihydroxyphenyl)prop-2-enoyl]oxy]-1,5-dihydroxycyclohexane-1-carboxylic acid), but not their interactions, we can assume that these metabolites are affected by cultivar similarly across both tissues. For example, metabolite S was slightly more abundant in ‘Record’ than ‘Quarta’ when considering the same tissues. While metabolite S showed an increase in abundance in small berries, metabolites C and R decreased in abundance from flower to big berry.

Some detected and assigned secondary metabolites were selected for quantification by HPLC-MS/MS based on the following criteria: (1) high peak intensity, (2) presence in almost all sample groups, (3) possible bioactivity, and (4) novelty in potato. This resulted in three phenolic compounds with possible economic value: metabolite P, kaempferol-3-*O*-galactoside and coniferin. Coniferin was present under the limit of quantification in all of the samples. However, metabolite P and kaempferol-3-*O*-galactoside were detected following the two-phase liquid–liquid extraction with MTBE and methanol. They were quantified in the polar phase, given their absence in the non-polar phase. Compared to the glycoalkaloid content, the levels of the phenolic compounds were much lower, not exceeding 1.43 mg/100 g FW (**Table 5**). Metabolite P (untargeted analysis peak intensities QF 5.00×10^7^, QSB 1.73×10^5^ and RF 1.93×10^7^) was only above the limit of quantification in flower samples, as shown in **Table 4** (negative values of normalized means). Similarly, kaempferol-3-*O*-galactoside, which was present in most flower and berry samples (untargeted analysis peak intensities QF 2.19×10^7^, QSB 1.12×10^6^, QBB 5.26×10^5^, RF 3.26×10^7^ and RSB 5.41×10^6^) was also more abundant in flowers based on HPLC-MS/MS analysis.

**Table 5:** HPLC-MS/MS quantified phenolic compounds present in potato tissues from plants cultivated in a greenhouse.

Cultivar	Tissue	Phenolic compounds (mg/100 g FW)	
		Metabolite P	Kaempferol-3-*O*-galactoside
‘Quarta’	Flower	1.21 ± 0.94	0.11 ± 0.001 a
	Small berry	<LoQ	0.05 ± 0.003 b
	Big berry	<LoQ	0.05 ± 0.003 b
‘Record’	Flower	1.43 ± 0.05	0.04 ± 0.06
	Small berry	<LoQ	<LoQ

Small berry: Ø < 1.5 cm. Big berry: Ø ≥ 1.5 cm. Different letters indicate significant differences between tissues for each compound within the same cultivar. Data were assessed by ANOVA followed by Tukey’s *post-hoc* test (p < 0.05) for ‘Quarta’ and are shown as means ± SD for n = 3 (<LoQ means that the peak detectable, but the signal/noise ratio is less than 10.). Metabolite P: 2,6-dihydroxy-2-[(4-hydroxyphenyl)methyl]-4-[(2S,3R,4S,5S,6R)-3,4,5-trihydroxy-6-(hydroxymethyl)oxan-2-yl]oxy-1-benzofuran-3-one.

### 3.4 Plant development changes in gene expression related to secondary metabolites

We analyzed the transcriptome of potato by-products by RNA-Seq to better understand how plant development changes affect gene expression regulating the accumulation of interesting secondary metabolites. The ‘Quarta’ and ‘Record’ tissues (flower, small berry and big berry) used for metabolomics analysis were also used for RNA-Seq. Pseudo-alignment of raw reads to the reference genome using Kallisto (Bray et al., 2016) revealed values of 69.6–81.6% (**Supplementary Table S7**). A complete transcript-level list of transcripts per million (TPM) across all RNA-Seq samples is provided in **Supplementary Table S8**.

PCA was performed on variance-stabilized counts to assess the clustering of samples across cultivars and tissues (**Figure 4**). Most of the variance (90%) was explained by PC1 and only 6% by PC2. Samples in the same group clustered together, with ‘Quarta’ flowers and big berries showing close clustering, whereas ‘Quarta’ small berries and all ‘Record’ samples showed a low level of within-group variability. However, like the metabolomics data, we observed a clear separation between flower and berry samples, indicating strong transcriptomic differences due to plant development.

**Figure 4:**
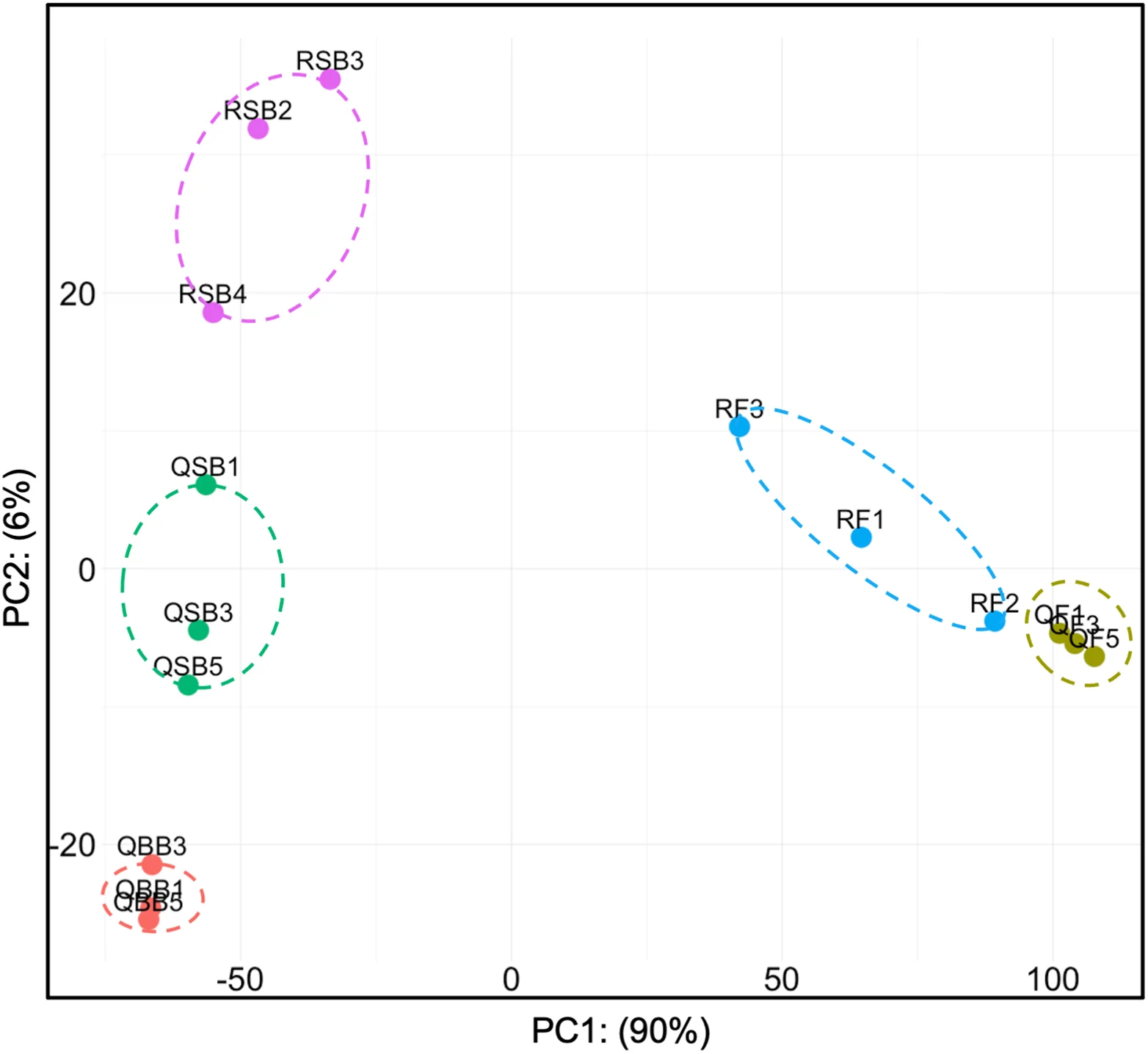
Principal component analysis (PCA) of variance-stabilized counts from RNA-Seq data. Q, ‘Quarta’; R, ‘Record’; F, flower; SB, small berry; BB, big berry. Color of dots and dashed circles = olive green, QF; teal, QSB; salmon, QBB; bright blue, RF; magenta, RSB.

Because cultivar ‘Record’ only produced small berries, we used DESeq2 to analyze the following contrasts: QF vs QSB, QF vs QBB, QSB vs QBB, RF vs RSB, QF vs RF and QSB vs RSB. A differential expression analysis was performed on 29,684 genes (Kallisto counts ≥ 10) with |log2-fold change (log2FC)| > 1 and adjusted p-values (padj) < 0.01 (**Figure 5**).

**Figure 5:**
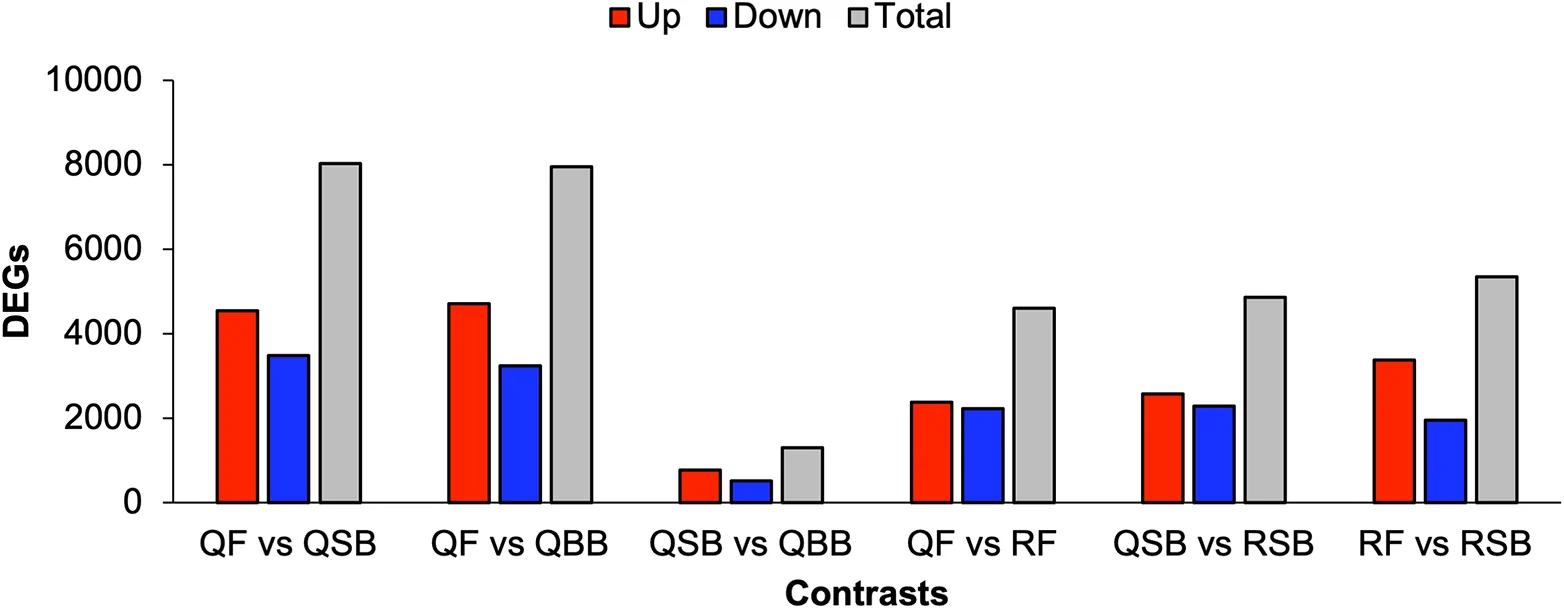
Differentially expressed genes (DEGs) for the comparisons QF vs QSB, QF vs QBB, QSB vs QBB, RF vs RSB, QF vs RF and QSB vs RSB. Filters: |log2FC| > 1 and padj < 0.01. Q, ‘Quarta’; R, ‘Record’; F, flower; SB, small berry; BB, big berry.

These results supported the PCA distribution, showing a larger number of differentially expressed genes (DEGs) for the variable tissue than the variable cultivar. The smallest number of DEGs was observed between small and big berries of the cultivar ‘Quarta’, indicating that the differences between these tissues are less than the differences between berries and flowers in general. The total number of DEGs in QF vs QSB (8021) and QF vs QBB (7952) was about six times higher than in QSB vs QBB (1300), with a larger number of upregulated genes than downregulated ones. In ‘Record’ samples, the total number of DEGs in RF vs RSB was lower (5344) than in ‘Quarta’. When the comparison was based on the same tissue of the two different cultivars, the number of DEGs recorded was 4612 in flowers (QF vs RF) and 2235 in small berries (QSB vs RSB), a smaller number of genes than in the flower-to-berry comparison (**Figure 5**).

Given that ‘Quarta’ produced all three tissues (flower, small berry and big berry), DEGs from the comparisons QF vs QSB, QF vs QBB and QSB vs QBB were used to determine how upregulated and downregulated genes are involved in the developmental transition from flower to the big berry. Venn diagrams were constructed to show the number of unique and common genes between the samples, revealing how potato plants remodel gene expression during development (**Figure 6**). Overall, many genes were switched on or off (3620 + 2113) when plants transition from flowers to berries, and most likely stay that way throughout berry development, with only 98 of the upregulated and 110 of the downregulated genes additionally regulated during ripening from small to big berries. Furthermore, 823 and 1257 genes were specifically regulated in flowers compared to small berries, whereas 769 and 810 genes were specifically regulated in flowers compared to big berries. A small number of genes (459 upregulated and 201 downregulated) were specifically regulated during ripening from small to big berries. The genes are listed in **Supplementary Table S9**.

**Figure 6:**
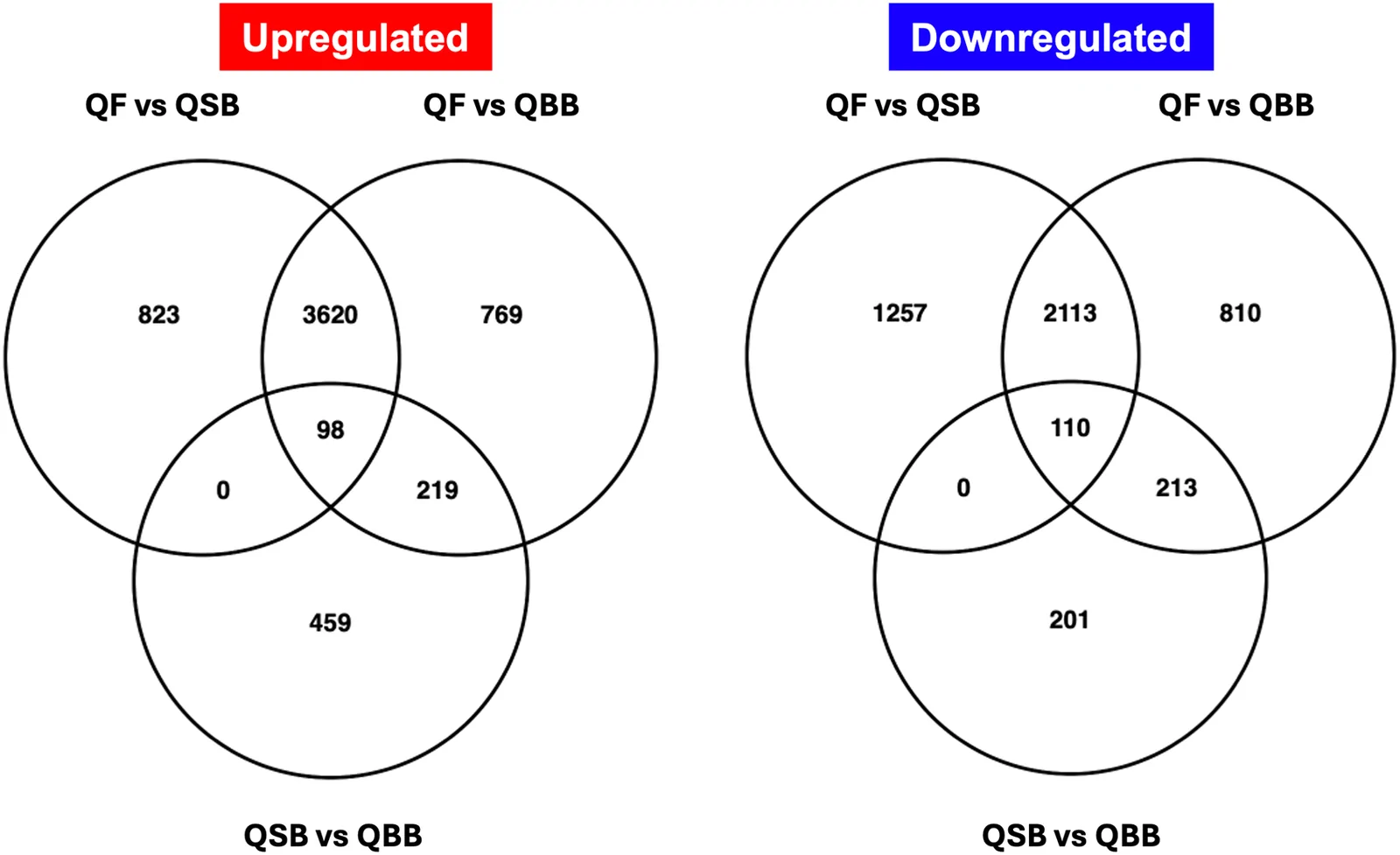
Venn diagrams based on lists of genes differentially expressed between ‘Quarta’ tissues.

Enrichment analysis was applied to genes commonly upregulated (3718) and downregulated (2223) in both flower-to-berry comparisons. For the upregulated genes, transcripts were assigned to 106 MapMan4 categories (Schwacke et al., 2019), with enrichment factors (EFs) of up to 7.62 (**Supplementary Table S8**). The top 30 processes upregulated in flowers included solute transport, reproduction and phytohormone action, indicating a positive shift from flower to berry. On the other hand, transcripts from downregulated genes represented 89 MapMan4 categories with EFs of up to 13.76 (**Supplementary Table S10**). The top 30 processes upregulated in berries included cell wall organization, enzyme-related processes and phytohormone action (e.g., auxin perception and signal transduction). **Supplementary Figure S5** shows the top 30 MapMan4 categories with an FDR cutoff of 0.05.

From the transcript lists provided by enrichment analysis, only upregulated genes in flower tissue presented ontologies related to the Mercator group “secondary metabolism” (EF = 2.03, FDR = 2.30×10^-5^, 84 transcripts in MapMan4 category corresponding to 56 genes). Among these genes, 39 belonged to the terpenoid biosynthesis (EF = 2.22, FDR = 7.98×10^-5^, 62 transcripts in MapMan4 category), of which 13 were part of the carotenoid biosynthesis (EF = 3.45, FDR = 9.24×10^-4^, 24 transcripts in MapMan4 category), and six were part of the carotene biosynthesis (EF = 4.31, FDR = 1.41×10^-2^, 13 transcripts in MapMan4 category). Other genes included in the “secondary metabolism” category were mostly related to the synthesis of phenylpropanoids, steroids, and other terpenoid branches, such as sesquiterpenes. Genes and their corresponding functions within the “secondary metabolism” MapMan4 category are listed in **Supplementary Table S11**. To link our metabolomic data to the RNA-Seq results, we analyzed the expression of genes within the MapMan4 category “secondary metabolism” (**Figure 7**).

**Figure 7:**
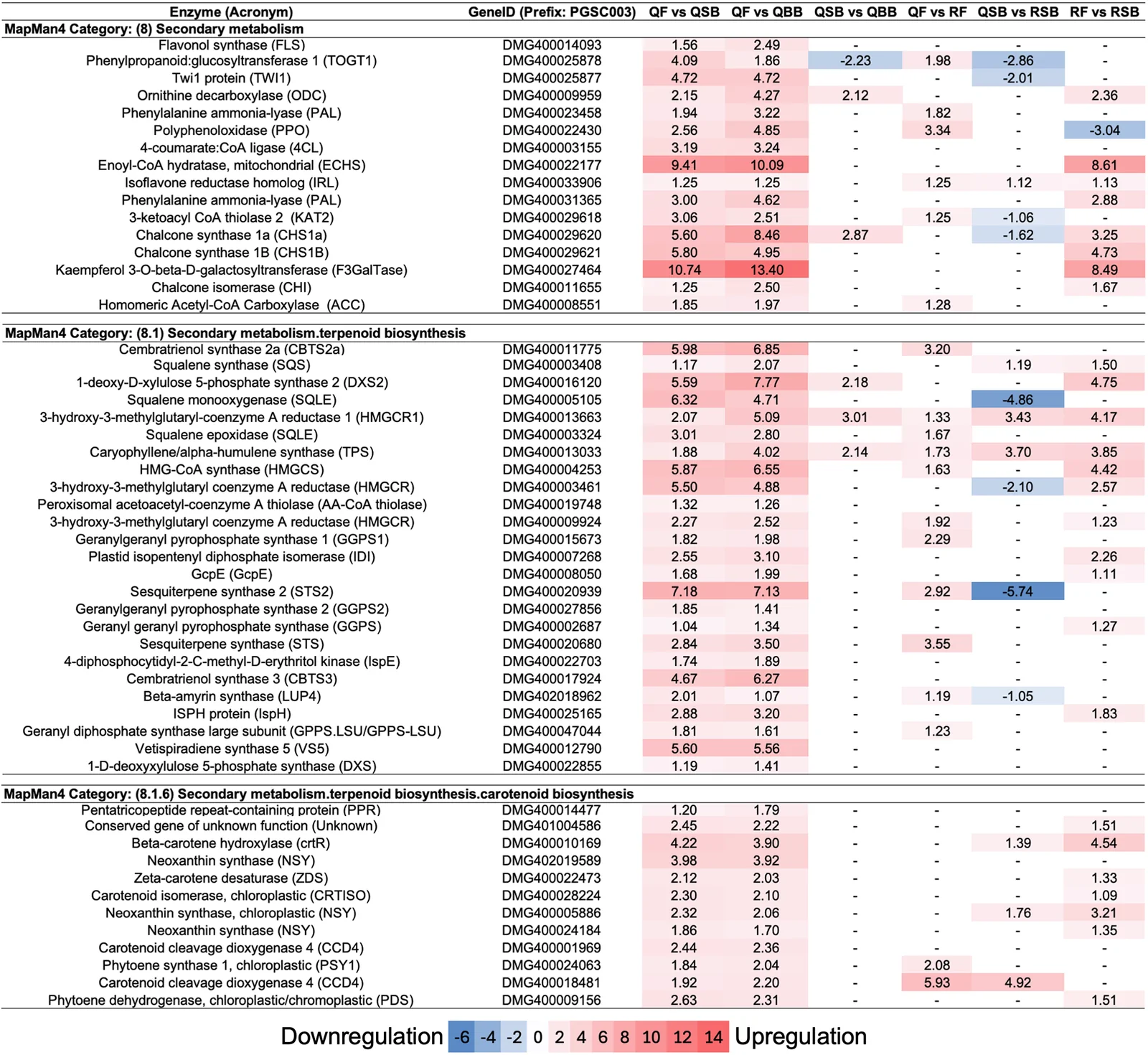
Expression (|log2FC| > 1, padj < 0.01) of genes included in MapMan4’s category “secondary metabolism”. Shades of red indicate upregulation, shades of blue indicate downregulation. Genes that did not significantly change between samples are identified with the symbol (–). Q, ‘Quarta’; R, ‘Record’; F, flower; SB, small berry; BB, big berry.

We identified 52 genes encoding 48 known enzymes involved in the biosynthesis of secondary metabolites such as phenylpropanoids, terpenoids, steroidal glycoalkaloids and carotenoids. Comparable to the tissue and cultivar specificities described above, the majority of genes with significant differences in expression were observed for comparisons between tissues (flower vs small berry vs big berry) whereas fewer genes changed in the same tissue from different cultivars (QF vs RF, QSB vs RSB), and between similar tissues in the same cultivar (QSB vs QBB). For example, only five of the 52 genes were significantly upregulated in small berries compared to big berries of ‘Quarta’: PGSC0003DMG400009959 (ornithine decarboxylase, ODC), PGSC0003DMG400013663 (3-hydroxy-3-methylglutaryl-coenzyme A reductase 1, HMGCR1), PGSC0003DMG400029620 (chalcone synthase 1a, CHS1a), PGSC0003DMG400016120 (1-deoxy-d-xylulose 5-phosphate synthase 2), and PGSC0003DMG400013033 (caryophyllene/α-humulene synthase, TPS), with log2-fold changes between 2.12 and 3.01.

Given the higher amounts of some phenolic compounds, such as flavonoids, aurones and phenolic acids in flowers, we took a further look at gene expression corresponding to the relevant enzymes. We observed a higher expression of phenylalanine ammonia-lyase (PAL) genes in flowers compared to berries, for PGSC0003DMG400031365 in both cultivars and for PGSC0003DMG400023458 in ‘Quarta’, reaching log2-fold changes of 1.94–4.62. Both genes were associated with higher TPM counts than the other PAL genes found, which showed a repression in flowers compared to berries, as observed in the phenylpropanoid pathway in **Figure 8**, also showing a repression of expression in flowers compared to berries.

**Figure 8:**
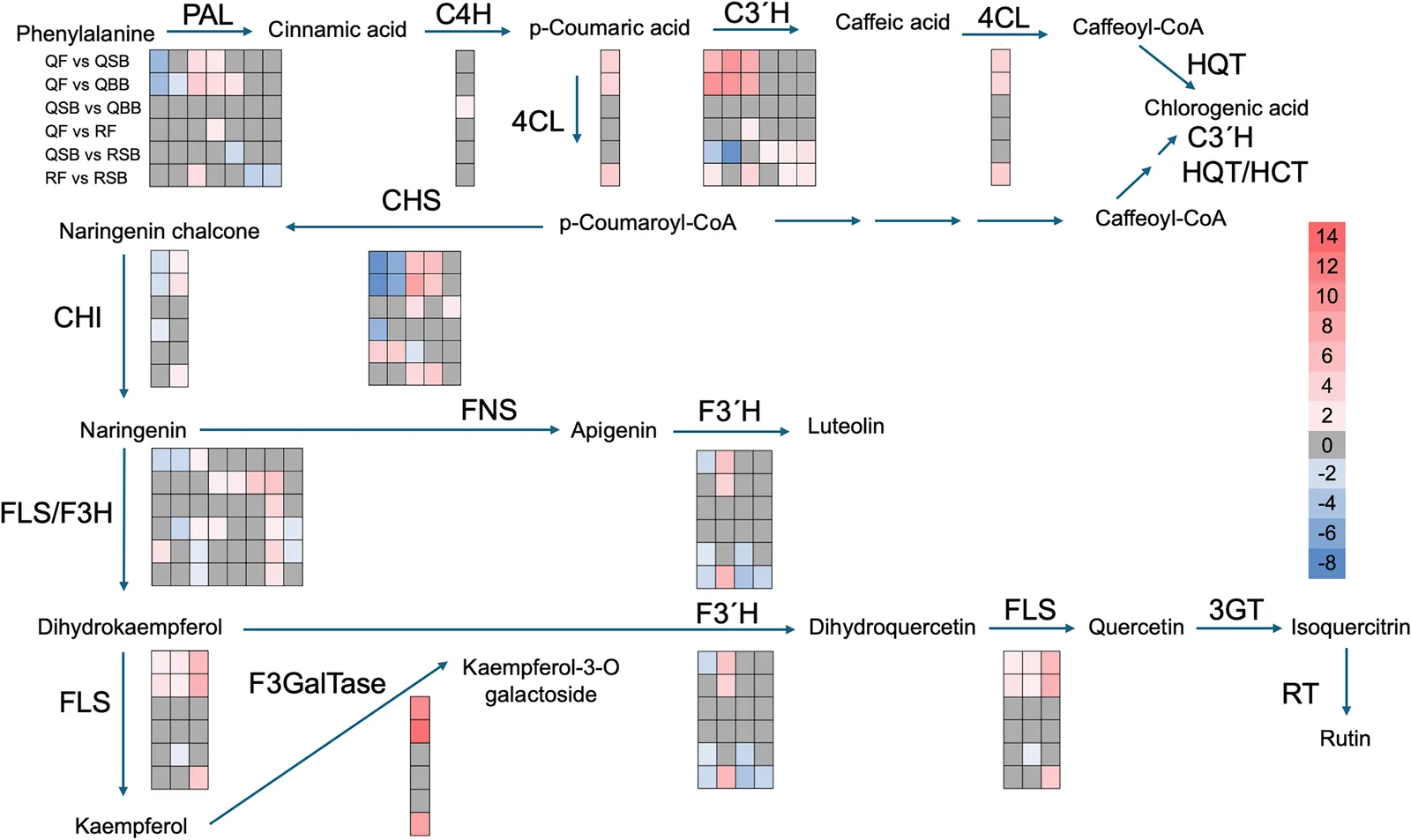
Phenylpropanoid biosynthesis pathway and fold changes in the expression of genes encoding the corresponding enzymes. Genes with |log2FC| > 1 and significant changes with padj < 0.01 are shown in color-coded intensities: red represents upregulation, blue represents downregulation. Rows show the different comparisons as stated in the upper-left corner. Columns are different genes counted from the left and explained in **Supplementary Table S12**. PAL, phenylalanine ammonia lyase; C4H: cinnamic acid 4-hydroxylase; 4CL, 4-coumarate-CoA ligase; C3′H, *p*-coumaroyl quinate/shikimate 3′-hydroxylase; HCT, hydroxycinnamoyl CoA shikimate/quinate:hydroxycinnamoyl transferase; HQT, hydroxycinnamoyl CoA quinate:hydroxycinnamoyl transferase; CHS, chalcone synthase; CHI, chalcone isomerase; FNS, flavone-synthase; F3H, flavanone-3-hydroxylase; F3′H, flavonoid-3′-hydroxylase; FLS, flavonol synthase; 3GT, flavonoid 3-*O*-glucosyltransferase; RT, 3-*O*-rhamnosyltransferase; F3GalTase, kaempferol 3-*O*-β-d-galactosyltransferase. The pathway is adapted and modified from Knollenberg et al. (2018), Choi et al. (2022), Muruganathan et al. (2022), and Yang et al. (2024).

Further along this pathway (**Figure 8**), we found a gene encoding 4-coumarate-CoA ligase (4CL) that was upregulated in flowers (PGSC0003DMG401009291). Genes encoding *p*-coumaroyl quinate/shikimate 3′-hydroxylase (C3′H), 4CL, chalcone synthase (CHS), chalcone isomerase (CHI), flavonoid-3′-hydroxylase (F3′H) and flavonol synthase were also upregulated in flowers, but other genes in the same pathway showed no changes between tissues or cultivars. Many phenolic compounds are glycosylated, and we observed the strong upregulation of a kaempferol 3-*O*-β-d-galactosyltransferase (F3GalTase) gene (PGSC0003DMG400027464) in flowers compared to berries with a log2-fold induction between 8.49 and 13.40.

In the context of terpenoids, we identified 25 DEGs encoding enzymes from the methylerythritol phosphate (MEP) and mevalonate (MVA) pathways (**Figure 9**). In both pathways, genes encoding the initial enzymes were strongly upregulated in flowers. From the MEP pathway, 1-deoxy-d-xylulose 5-phosphate synthase 2 (*DXS2*) (PGSC0003DMG400016120) showed a log2-fold change of 5.59 compared to small berries, and 7.77 compared to big berries. From the MVA pathway, 3-hydroxy-3-methylglutaryl-coenzyme A reductase (*HMGCR*) (PGSC0003DMG400003461) showed a log2-fold change of 5.50 compared to small berries and 4.88 compared to big berries. Genes encoding enzymes involved in squalene synthesis were also strongly upregulated in flowers. Different functional classes (squalene synthase/monooxygenase/epoxidase) presented log2-fold changes varying from 1.17 to 6.32.

**Figure 9:**
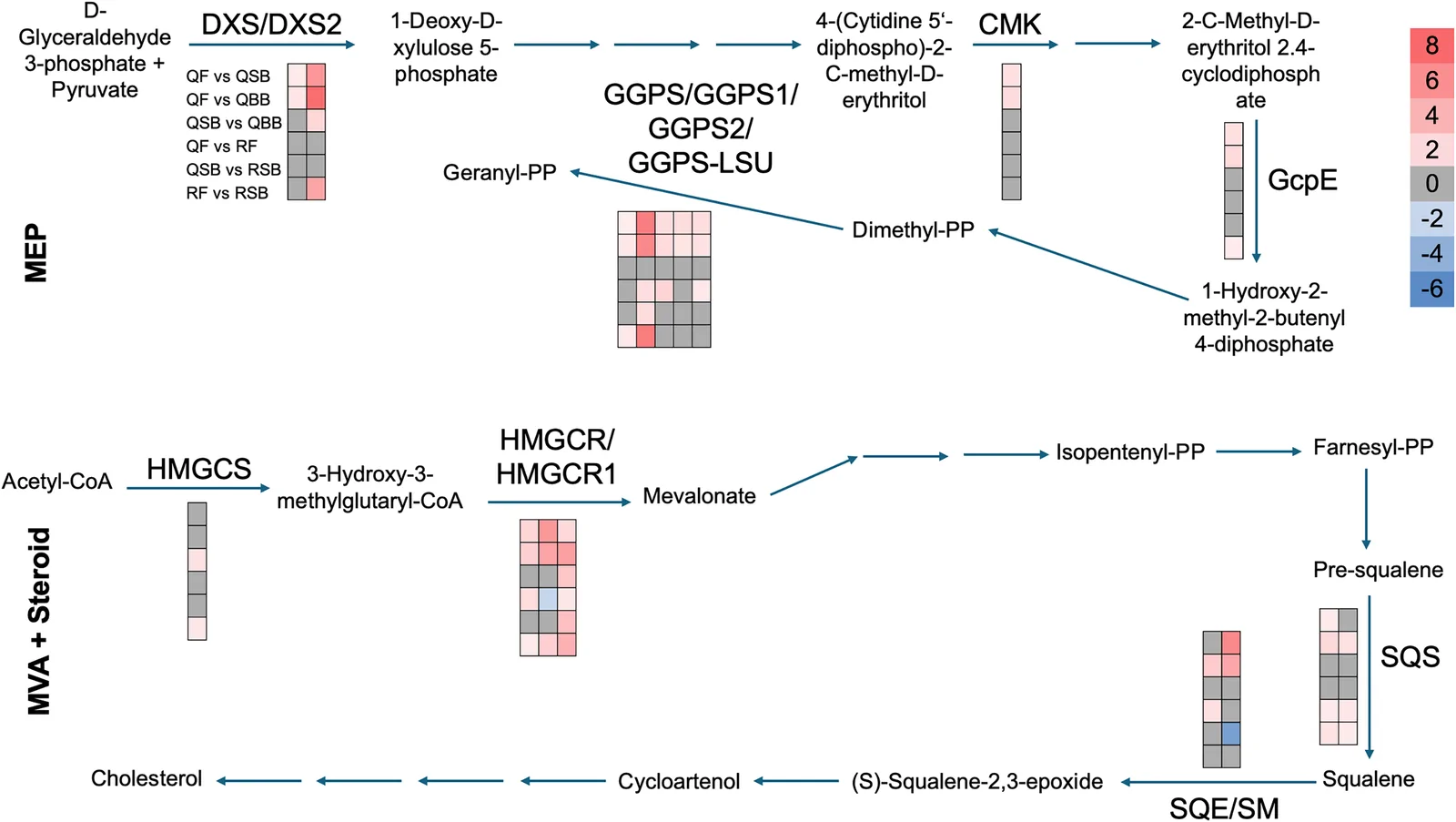
Terpenoid and cholesterol (steroid) biosynthesis pathways and fold changes in the expression of genes encoding the corresponding enzymes. Genes with |log2FC| > 1 and significant changes with padj < 0.01 are shown in color-coded intensities: red represents upregulation, blue represents downregulation. Rows show the different comparisons as stated in the upper left corner. Columns are different genes counted from the left and explained in **Supplementary Table S12**. DXS, 1-deoxy-d-xylulose 5-phosphate synthase; CMK, 4-diphosphocytidyl-2-C-methyl-d-erythritol kinase; GGPS, geranylgeranyl pyrophosphate synthase; GGPS-LSU, geranyl diphosphate synthase large subunit; HMGCS, HMG-CoA synthase; HMGCR, 3-hydroxy-3-methylglutaryl-coenzyme A reductase; SQS, squalene synthase; SQLE, squalene epoxidase; SM, squalene monooxygenase. The pathway is adapted and modified from Kollas et al. (2002); Benakanakere et al. (2014); Xie et al. (2017); Mani et al. (2021), and Nahar et al. (2017).

Interestingly, genes encoding enzymes involved in downstream steroidal glycoalkaloid formation, such as GAMEs/SGTs/DPS, were not significantly changed in our samples. However, the PGSC0003DMG400013663 gene, encoding the upstream and rate-limiting enzyme 3-hydroxy-3-methylglutaryl-coenzyme A reductase 1 (HMGCR1), was upregulated in flower and small berry tissues regardless of cultivar. Moreover, the log2-fold change ranged from 1.33 to 5.09, with more upregulation in flowers compared to big berries.

Regarding carotenoids, genes were differentially expressed not only at the beginning of the pathway but also in the branches for the formation of carotenes and xanthophylls. Overall, the genes in the carotenoid pathway were mostly upregulated in flowers of both cultivars, but the log2-fold change values were low compared to the other pathways described above. The expression of genes encoding early enzymes such as phytoene synthase 1 (PSY1), phytoene dehydrogenase (PSD), ζ-carotene desaturase (ZDS), and carotenoid isomerase (CRTISO), which catalyze the conversion of GGPP into lycopene, ranged from 1.09 to 2.63. However, downstream enzymes such as β-carotene hydroxylase (crtR) and neoxanthin synthase (NSY) showed log2-fold changes up to 4.54.

We also observed changes in the expression of a larger fraction of soluble sulfotransferase (SOT) genes, here annotated as flavonol 4′-sulfotransferases (**Figure 10**). In the small and big berries of ‘Quarta’, 12–13 genes of this family are upregulated with log2-fold changes of 1.5–8.2 compared with flowers, with a stronger upregulation in big berries for some genes, significant for *SOT16* in QSB × QBB. This expression change was not observed for the flower-to-berry comparison in ‘Record’, where *SOT05* and *SOT20* were upregulated in flowers instead. ‘Quarta’ SOT genes showed lower expression in flowers and higher expression in berries, which might reduce the overall difference between the tissues.

**Figure 10:**
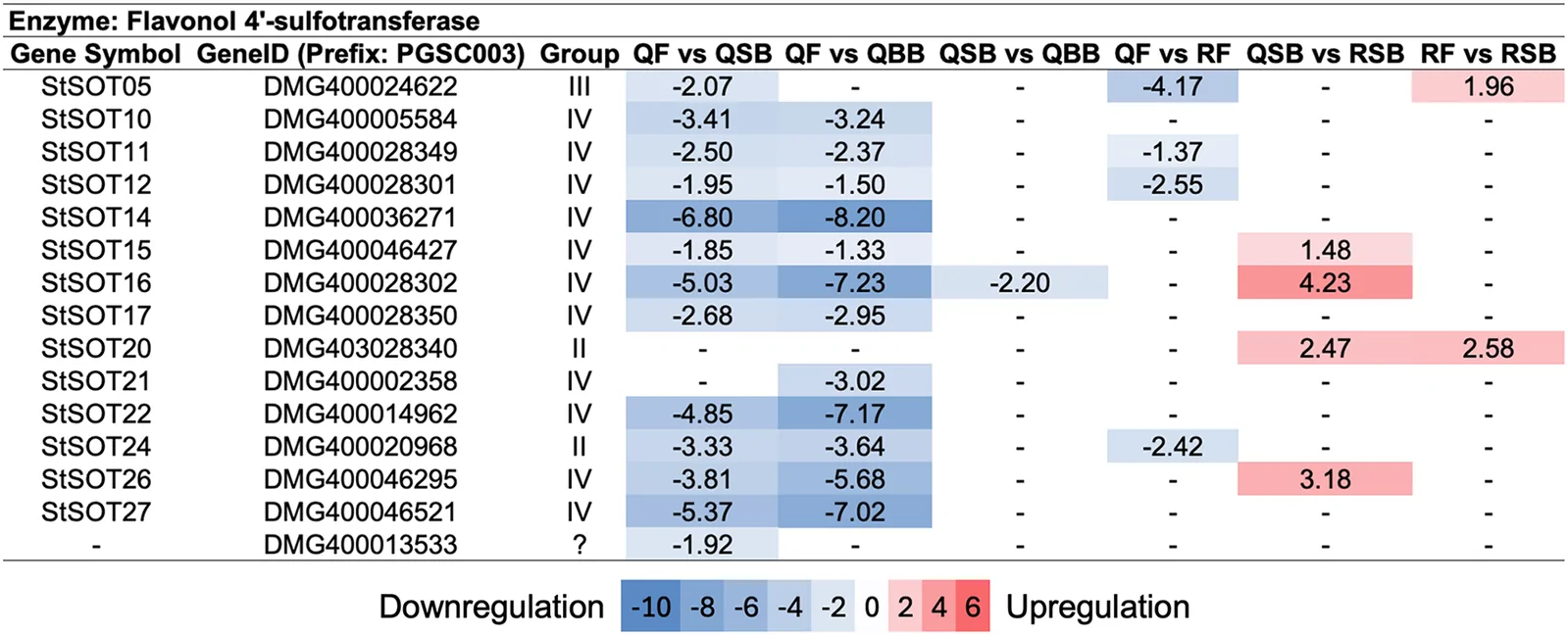
Expression (|log2FC| > 1, padj < 0.01) of genes encoding flavonol 4′-sulfotransferases. Gene symbols and groups I–IV were classified as described by Faraji et al. (2021). Shades of red indicate upregulation, shades of blue indicate downregulation, and genes with no changes are identified with (–). Q, ‘Quarta’; R, ‘Record’; F, flower; SB, small berry; BB, big berry.

## 4 Discussion

The use of potato processing waste as by-products for alternative processes has received a great deal of attention, whereas agricultural by-products like flowers and berries have been largely overlooked, despite being rich in bioactive secondary metabolites and suitable for a circular bioeconomy (Bueno da Silva et al., 2024). We therefore combined analytical chemistry with the transcriptomic and metabolomic analysis of potato flowers and berries to understand the potential utilization of these tissues for extraction of plant special metabolites which might be of use for the pharmaceutical and plant protection sectors, possibly providing opportunities for value addition to this material. We investigated the impact of early flower and berry harvesting on tuber yield. Flowers and berries are not required for potato propagation because this is achieved using seed potatoes. However, flowers and berries may affect tuber yields by competing for photosynthates (Bartholdi, 1942; Jansky and Thompson, 1990), especially in cultivars with high flower and fruit production. The removal of flowers was previously shown to significantly increase the yield by 20–40% in the first year but did not influence yields in the subsequent year (Jansky and Thompson, 1990).

With average tuber yield varying between 15.79 and 22.51 t/ha in 2022 and 35.07 and 45.65 t/ha in 2023, the yields of our two cultivars were lower than the average recorded in Germany in the same years (40.11 and 43.85 t/ha, respectively) (Destatis, 2026), which may be a cultivar-related effect. When comparing the treatments, early flower harvesting did not affect the tuber yield of ‘Quarta’ or ‘Granola’ plants in field trials. Harvesting berries generally had no effect on tuber yields either, with one exception: harvesting berries before the end of the crop cycle significantly reduced the tuber yield of ‘Quarta’ plants in our second field trial (28.88 t/ha). Given this decrease only happened in one of the two cultivars and only in 2023, we assume this is an isolated event.

Overall, the field results showed a variation in by-product and tuber yields between the two field trials. For the by-products, this was more pronounced in ‘Quarta’ plants, with an increase in flower production in 2023, whereas berry production was reduced for both cultivars in the second year. This difference was more subtle for tuber yield, although noticeable that 2023 performed better than the previous year (**Figure 2**). This may be attributed to different environmental conditions particular to each year. Moreover, plots that composed the experimental area of 2023 were built in a different area within the same site (Campus Klein Altendorf) and received an extra border, which could have also benefited the plants to thrive more than the previous year. This, allied to the reduction in tuber yield in the berry treatment for ‘Quarta’ cultivar, shows that a third field trial could have helped to achieve a better conclusion. Therefore, further field experiments also with more different cultivars should be performed to exclude any impact of side stream harvesting on tuber yield.

We quantified well-known compounds such as potato glycoalkaloids in flowers and berries from field and greenhouse cultivation sites. Potato glycoalkaloids, although toxic to humans (Akinboye et al., 2023), have pharmaceutical properties and may also be useful for plant protection (Benkeblia, 2020). For example, potato peel extracts rich in glycoalkaloids reduced cytokine production by ~22% in rat models of inflammation (Kenny et al., 2013) and α-chaconine in particular suppressed the activity of stomach and liver cancer cells by up to 94.9% and 89.7%, respectively (Friedman et al., 2005). Furthermore, we previously showed that α‐chaconine inhibits the growth and survival of plant parasites and pathogens, such as the nematode *Heterodera schachtii* by 63% and the fungus *Leptosphaeria maculans* by more than 70% (Bueno da Silva et al., 2025).

Due to toxicity in humans, potato breeding programs focus on reducing the quantity of glycoalkaloids in edible tubers (Love et al., 1996; Esposito et al., 2002) but the non-edible part of the plant nevertheless maintains a high TGC (defined here as the sum of α-solanine and α-chaconine) for defense and resistance (Friedman, 2006), with most accumulating in tissues with high metabolic activity (Friedman et al., 1997). Our results confirmed a higher TGC in flowers than other tissues, including berries. The TGC of flowers harvested from the greenhouse varied between 393.95 and 485.80 mg/100 g FW, depending on the cultivar. Kozukue et al. (1987) also reported a higher glycoalkaloid content in flowers (571.15 mg/100 g FW in calyxes, 497.22 mg/100 g FW in petals) than leaves (35.95–58.69 mg/100 g FW) and shoots (7.1 mg/100 g FW). Friedman and Dao (1992) later demonstrated the differential accumulation of glycoalkaloids in potato tissues with the following ascending order: tubers (14.62 mg/100 g FW) < main stems < small stems < roots < leaves < sprouts (997.06 mg/100 g FW). Berries originating from breeding programs could be placed between main and small stems, with a content of ~40 mg/100g FW. We observed a dilution effect from flowers to berries, but the further development of the berry did not change the TGC so drastically.

Although flowers have a higher TGC, the fresh mass of berries led to higher amounts of extractable glycoalkaloids. For instance, berries from ‘Granola’ cultivar provided 4 to 5 times more TGC than flowers in 2022 (flowers: 66.01 g TGC/ha; berries: 260.00 g TGC/ha) and 2023 (flowers: 10.33 g TGC/ha; berries: 54.19 g TGC/ha), respectively. In 2022 ‘Quarta’ berries (284.09 g TGC/ha) also provided ca. 3 times more TGC than flowers (105.30 g TGC/ha). However, in the following year, flower tissues from ‘Quarta’ provided 122.64 g TGC/ha, while berries only provided 41.05 g TGC/ha. The extractability of glycoalkaloids from berries was confirmed albeit with lower purity compared to flowers, almost comparable to extraction from leaves (Stadler et al., 2025). Improving extraction methods might help to fully harness the glycoalkaloids from potato berries. Optimized protocols for the extraction of glycoalkaloids from agricultural side-streams (leaves or berries) would offer an environmentally friendly alternative to a semi-synthesis (Stadler et al., 2025).

We did not detect other alkaloids in our flower and berry samples, such as the aglycones solanidine and solasodine, or the glycoalkaloid α-solamargine. The lack of solanidine may reflect the efficient conversion of solanidine to α-solanine and α-chaconine (Friedman, 2006). Solasodine and α-solamargine have been quantified in potato leaves and tubers (Baur et al., 2022) and in other solanaceous berries (Al Sinani and Eltayeb, 2017; Laware and Shirole, 2022; Lucier et al., 2024; Panchal and Shah, 2025), and are also usually present in eggplant (*Solanum melongena*) (Sánchez-Mata et al., 2010; Cham, 2011) and in the tubers and leaves of wild *Solanum* plants (Distl and Wink, 2009). Although we were unable to detect them in potato, the α-chaconine/α-solanine ratios we observed (1.14–3.33-fold more α-chaconine) are similar to previous reports. In potato flesh, peel and tuber tissues, ratios of 1.4–2.6-fold more α-chaconine have been recorded (Friedman et al., 2003). This suggests that, regardless of the tissue type or cultivation site, α-chaconine is the major potato glycoalkaloid, and perhaps the most readily extractable alkaloid. Extreme ratios of 17-fold α-chaconine were observed by Şengül et al. (2004) in the cultivar ‘Granola’ when studying the impact of light and wounding on tubers and sprouts, which could stimulate the accumulation of glycoalkaloids. Modifying the content of specific metabolites by such treatments can increase the value of biomass, as discussed also for other side-streams (Junker-Frohn et al., 2019; Genzel et al., 2021).

Although α-solanine and α-chaconine are the major glycoalkaloids in potato tissues (Liu et al., 2024), we also detected minor glycoalkaloids (solanandaine and dehydrosolanine) in flowers and berries from both cultivars. Solanandaine is also detected in eggplant (Lelario et al., 2019), ripe fruits of wolf apple (*Solanum lycocarpum*) (Morais et al., 2020), and green fruits of *Solanum asperum* (Silva et al., 2008). Solanandaine was the only tissue-specific glycoalkaloid in our study and became less abundant during development. Dehydrogenated forms of glycoalkaloids, such as dehydrosolanine, are common as traces in wild *Solanum* species (Väänänen et al., 2005). In cultivated potato, dehydrosolanine and also dehydrochaconine were previously detected in leaves (Carvajal-Acosta et al., 2025), sprouts, and the periderm and medulla of tubers (Razgonova et al., 2023; Deng et al., 2021). In our samples, solanandaine and dehydrosolanine were detected in all flowers and small berries of ‘Quarta’ and ‘Record’ cultivars, as well as ‘Quarta’ big berries, but high peak intensity (> 1.0×10^8^) was only observed for solanandaine in flowers.

High concentrations of secondary metabolites are required for extraction, so we focused on those with the highest peak intensities. For example, a strong peak (> 1.0×10^9^) was observed for *N*-methylnicotinic acid (trigonellin) in all groups and cultivars. This is a pyridine alkaloid found at high levels in the seeds of clover (*Trifolium incarnatum*) (58 µmol/g FW), coffee (*Coffea arabica*) (53 µmol/g FW), soybean (*Glycine max*) (1.2 µmol/g FW) and rice (*Oryza sativa*) (0.04 µmol/g FW) (Ashihara et al., 2015). In some trigonellin-accumulating plants, like the four o’clock flower (*Mirabilis jalapa*) and coffee, trigonellin is also detected in flowers and other organs (Ashihara and Watanabe, 2014). We observed a slight decrease in peak intensity from flower (Q 3.42×10^9^, R 5.51×10^9^) to small berry (Q 2.96×10^9^, R 3.61×10^9^), with a further increase in big berries, when present (Q 4.15×10^9^), indicating that it might accumulate in flowers and further during seed formation/maturation in the berries. The properties of this alkaloid have been described extensively in the context of neuroprotection and diabetes (Liang et al., 2023; Nguyen et al., 2024).

The presence of phenolic compounds in potato by-products could be an advantage for their utilization because phenolic acids and polyphenols have strong antioxidant activity (Kruk et al., 2022; Chen et al., 2024). Well-known compounds detected in ‘Quarta’ and ‘Record’ cultivars included chlorogenic acid, neochlorogenic acid and rutin, some of the major phenolic compounds in potato tissues (Mäder et al., 2009; Akyol et al., 2016; Ru et al., 2019), and chlorogenic acid has been detected in potato berries before (Devkota et al., 2015). The intensity peaks of chlorogenic acid and neochlorogenic acid were higher in small berries than in flowers for both cultivars, and higher in ‘Quarta’ small berries than big berries, whereas rutin showed a similar intensity peak in flowers and small berries (~1.0×10^8^). Rutin has been detected in potato flowers earlier, with amounts reaching up to 10% of the tissue (Bandyukova and Sergeeva, 1974). The option of rutin extraction from agricultural/horticultural side-streams has been investigated before (Junker-Frohn et al., 2019). Interestingly, in ‘Quarta’, the intensity peak of rutin decreased to ~1×10^6^ in big berries. Usenik et al. (2015) also observed a higher accumulation of neochlorogenic acid and rutin in smaller fruits when studying sweet cherry (*Prunus avium*). In our study, only neochlorogenic acid and rutin showed significant tissue specificity.

Other compounds with a large variation in intensity between flower and berry samples included metabolite P (2,6-dihydroxy-2-[(4-hydroxyphenyl)methyl]-4-[(2S,3R,4S,5S,6R)-3,4,5-trihydroxy-6-(hydroxymethyl)oxan-2-yl]oxy-1-benzofuran-3-one), which is a flavonoid and more specifically an aurone (Yoshikawa et al., 1998). Aurones possess polyhydroxylated benzofuran ring structures that are easily converted into bioactive derivatives with antioxidant (ROS scavenging), anticancer, antibacterial and antifungal activities, and even insecticidal activity against *Spodoptera litura* larvae (Mazziotti et al., 2021; Lazinski et al., 2022). Therefore, the abundance of this metabolite in potato flowers (‘Quarta’ 1.21 mg/100 g FW; ‘Record’ 1.43 mg/100 g FW) might enhance the benefits of exploring this material for commercial uses.

Thus far, to our knowledge, potato flowers and berries have not been investigated using an untargeted metabolomics approach, so their specific accumulation profiles and potential utilization as a source of secondary metabolites have been overlooked. We found that secondary metabolites with potential pharmaceutical and agricultural applications have both tissue specific and developmentally regulated profiles. RNA-Seq analysis revealed a clear division between the transcriptomes of potato flowers and berries. For example, flowers presented a larger number of upregulated genes compared to small and big berries (the latter only for ‘Quarta’) whereas comparisons between the same tissue in different cultivars (e.g., QF vs RF, QSB vs RSB) or between berries at different ripening stages (QSB vs QBB) revealed fewer DEGs. The impact of different cultivars or similar tissues at different stages is therefore minimal compared to different tissues. This agrees with Ochoa-Alejo et al. (2024) when studying 12 cultivars of chili pepper (*Capsicum annuum* L.), where differences between cultivars were narrower than differences between tissues, indicating that the developmental effect from flowers to fruits is indeed responsible for greater transcriptomic complexity.

Enrichment analysis showed that genes upregulated in flower tissue compared to berry tissue at both developmental stages were strongly related to solute transport. This is consistent with the metabolic demand of this tissue for further fertilization processes (Ruan et al., 2012). From flower to berry formation, we observed a shift in the enrichment analysis, where upregulated genes in berries were related to cell wall organization and cell expansion, reflecting fruit set, growth and development. Similar findings were reported by Giovannoni (2004) and Karlova et al. (2014) when studying early fruit growth in tomato (*Solanum lycopersicum*). This genome-wide expression analysis also facilitates the understanding of differences in flower and berry metabolism beyond the results of metabolomics, such as the observed significant enrichment of modulated genes in the terpenoid and carotene pathways, among others. Important and rate-limiting enzymes for the synthesis of phenolic compounds, terpenoids and steroidal glycoalkaloids were modulated in their gene expression. In the phenylpropanoid pathway, genes encoding upstream enzymes like C3′H and 4CL were upregulated in flowers. These have a role in the conversion of *p*-coumaric acid to chlorogenic acid (Lindl et al., 1973; Kühnl et al., 1987), a compound that was present in the potato flower/berry metabolome but did not accumulate in flowers accordingly. As we did not detect changes expression of HQT, hydroxycinnamoyl CoA quinate:hydroxycinnamoyl transferase, further metabolism of caffeoyl-CoA might occur into flavonoids or into lignin biosynthesis might also occur (Chen et al., 2026). Moreover, genes encoding downstream enzymes like F3GalTase were also upregulated in flowers. F3GalTase converts kaempferol to kaempferol-3-*O*-galactoside (Miller et al., 1999). The high log2FC observed for this compound might explain why this compound was found at higher amount in flowers and not in berries.

Enrichment analysis also specifically highlighted the presence of genes related to the terpenoid biosynthetic pathway, especially among the genes upregulated in flowers. Flowers tend to accumulate monoterpenes and sesquiterpenes as volatile compounds for attraction during pollination/fertilization (Pichersky and Gershenzon, 2002; Dudareva et al., 2004). The upregulated genes included representatives from the two main branches of terpenoid biosynthesis (the MEP and MVA pathways), as well as more specific terpenoid derivatives. For the MEP pathway, *DXS* was strongly upregulated in flowers compared to big berries. DXS catalyzes the first step of this pathway, which converts pyruvate and glyceraldehyde-3-phosphate into 1-deoxy-d-xylulose-5-phosphate (Tian et al., 2022). For the MVA pathway, the rate-limiting enzyme is HMGCR, converting 3-hydroxy-3-methylglutaryl-coenzyme A into mevalonate (Wang et al., 2023). Further down the MVA pathway, genes encoding important enzymes for the formation of sesquiterpenes and triterpenes (STS, SQS and SQLE) were mostly only upregulated in flowers, regardless of the cultivar.

Although specific genes engaged in glycoalkaloid metabolism (GAME) were not differentially expressed in our samples, genes encoding indicative enzymes in the MVA and steroid pathways were upregulated in flowers. This is because the MVA pathway is upstream of the production of steroidal glycoalkaloids (Liu et al., 2024). For example, HMGCR1 catalyzes the initial conversion of HMG-CoA (3-hydroxy-3-methylglutaryl-coenzyme A) into mevalonate (Gesto et al., 2020). The second phase involves squalene-related enzymes converting farnesyl pyrophosphate (FPP) into squalene, which, through cycloartenol, forms cholesterol, the precursor of steroidal glycoalkaloid biosynthesis (Cárdenas et al., 2016; Coman et al., 2018; Liu et al., 2024).

Transcriptomics also revealed the differences of genes related to further plant secondary metabolites not measured or identified in the metabolomics. Sulfotransferase (SOT) genes were also differentially expressed in both cultivars. These enzymes, transfer sulfate groups to several classes of substrates. They use 3′-phosphoadenosine 5′-phosphosulfate (PAPS) as a sulfuryl donor and modify the solubility and biological activity of secondary metabolites in water (Klein and Papenbrock, 2008; Hirschmann et al., 2014). We found that SOT genes were strongly upregulated in berries compared to flowers, which may modulate defense and/or fruit ripening. For example, upregulation in ‘Quarta’ big berries compared to small berries was also observed for the gene PGSC003DMG4000282. A higher number of sulfonated metabolites might be found in berries compared to flowers; such compounds were however, not detected or identified in the performed metabolomic analysis.

## 5 Conclusions

We have evaluated the potential utilization of potato by-products as an innovative source of bioactive compounds as a basis for future development of pharmaceutical and/or plant protection products. Overall, our results showed that non-conventional tissues, such as flowers and berries, also contain interesting metabolites with useful biological activities. We observed the presence of glycoalkaloids, phenolic compounds and terpenoids that accumulated differently according to the tissue, without a substantial influence of the cultivar. Looking at the advantages of each tissue, flowers contained a larger amount of glycoalkaloids than berries (independent of berry size), the largest portion being α-chaconine. Also, the premature harvest of flowers did not influence the final tuber yield, and the harvesting of berries only reduced the yield for one cultivar in one year. However, berries have a much higher biomass yield than flowers and a more robust morphology, which not only facilitates harvesting but also improves shelf-life. Phenolic compounds already extensively described in potato (chlorogenic acid, neochlorogenic acid and rutin, among others) were also present in our samples, but we also identified compounds that, to our knowledge, have not been described in potato before and could add value to the flowers and berries. One example was the aurone metabolite P in potato flowers, which inhibits pathogens of plants and humans.

Considering the low scientific interest in the applicability of potato flowers and berries from commercial fields, our research aimed to expand the basic knowledge on these tissues and to suggest new sources of metabolites with potential utilization in a circular bioeconomy beyond those found in potato peel. It is understandable that this study presented a few limitations, such as the need for more field trials and a broader number of cultivars. To briefly clarify, the low number of cultivars was also due to the lack of berry production among the cultivars we tested in the background. However, even with this low number of cultivars, our results showed that the metabolome and transcriptome of flowers and berries showed that plant development plays a role in secondary metabolite profiles, so particular tissues should be prioritized according to the demand for specific metabolites.

Based on our main results, we see the use of potato berries as a source of α-chaconine as an advantage due to the high availability of this compound in this tissue and the strong reported bioactivity. With larger biomass and higher resistance to handling and storage, this tissue could allow scalability of extraction and better shelf life, which are important points to be further studied when there is interest in industrial applications. Future research could also explore potato flowers and berries as sources of more specific phenolic compounds, possibly helping to increase the value of this plant material for industry and also for farmers.

## Data Availability

The datasets presented in this study can be found in online repositories. The names of the repository/repositories and accession number(s) can be found below: https://www.ebi.ac.uk/ena, PRJEB115950; https://doi.org/10.60534/448zd-4hn11.
