# Supplementary material for: Golden biomass: unlocking the potential of neglected potato (Solanum tuberosum L.) side-streams as sources of bioactive compounds
Source: Front Plant Sci. 2026 Oct 5;17:1957940. doi: 10.3389/fpls.2026.1957940 (PMC13693557; doi:10.3389/fpls.2026.1957940)
Supplement: Supplementary file 1 [file DataSheet1.docx]

Supplementary Material

**Supplementary Figure S1.** Randomized block design (RBD) of the field trial conducted in 2022.

**Supplementary Figure S2.** Randomized block design (RBD) of the field trial conducted in 2023.

**Supplementary Figure S3.** Chemical structures of the compounds targeted for quantification in this study.

**Supplementary Figure S4.** Temperature range (°C) during plant cultivation under greenhouse conditions. Minimum and maximum temperatures are indicated in the graph. Time span: May to July 2022.

**Supplementary Table S1.** Mass spectrometry specifications for glycoalkaloid quantification.

| **Compound** | **Transition (m/z)** | **Fragmentation voltage (V)** | **Collision energy (eV)** |
| --- | --- | --- | --- |
| α-solanine | 868 → 398 | 250 | 80 |
|  | 868 → 96 |  |  |
| α-chaconine | 852 → 707 | 250 | 80 |
|  | 852 → 98 |  |  |
| α-solamargine | 868 → 851 | 250 | 65 |
| Solasodine | 414 → 397 | 150 | 40 |
|  | 414 → 294 |  |  |
| Solanidine | 398 → 98 | 150 | 55 |
|  | 398 → 56 |  |  |

**Supplementary Table S2.** Coefficient of determination R^2^, limit of detection (LoD) and quantification (LoQ), precision and accuracy for glycoalkaloid quantification.

| **Compound** | **R^2^** | **LoD**  **[nM]** | **LoQ**  **[nM]** | **Precision**  **[%]** | **Accuracy***  **[%]** |
| --- | --- | --- | --- | --- | --- |
| α-solanine | 0.9991 | 0.008 | 0.025 | 1.6 | -7.9 |
| α-chaconine | 0.9971 | 0.01 | 0.04 | 1.3 | -3.5 |
| α-solamargine | 0.9982 | 0.02 | 0.05 | 1.9 | -6.7 |
| Solasodine | 0.9976 | 0.005 | 0.02 | 0.8 | -1.8 |
| Solanidine | 0.9986 | 0.005 | 0.02 | 0.9 | 0.9 |

* Average of the accuracies of all calibration levels (2.5, 5, 10, 20, 35, 50, 75, 100 nM)

**Supplementary Table S3.** Mass spectrometry specifications for phenolic compound quantification. Metabolite P: 2,6-dihydroxy-2-[(4-hydroxyphenyl)methyl]-4-[(2S,3R,4S,5S,6R)-3,4,5-trihydroxy-6-(hydroxymethyl)oxan-2-yl]oxy-1-benzofuran-3-one.

| **Compound** | **Transition (m/z)** | **Cone voltage (V)** | **Collision energy (eV)** |
| --- | --- | --- | --- |
| Kaempferol-3-O-galactoside | 449.0 → 287.1  449.0 → 153.0 | 32 | 10  46 |
| Coniferin | 365.0 → 185.0  365.0 → 202.2 | 6 | 18 |
| Metabolite P | 473.0* | 54 |  |
| * determined in single ion reaction (SIR) mode | | | |

**Supplementary Table S4.** Coefficient of determination R^2^, limit of detection (LoD) and quantification (LoQ), precision and accuracy for phenolic compound quantification.

| **Compound** | **R^2^** | **LoD**  **[nM]** | **LoQ**  **[nM]** | **Precision**  **[%]** | **Accuracy***  **[%]** |
| --- | --- | --- | --- | --- | --- |
| Kaempferol-3-O-galactoside | 0.9995 | 2.0 | 6.6 | 1.2 | -1.2 |
| Coniferin | 0.9958 | 3.5 | 11.7 | 1.7 | 1.6 |
| Metabolite P | 0.9978 | 14.3 | 47.6 | 1.1 | -9.0 |

* Average of the accuracies of all calibration levels (0.5, 1, 1.5, 2.5, 5, 10, 20, 50 µM)

**Supplementary Table S5.** Peaks detected by untargeted metabolomic analysis based on reversed-phase ultra-performance liquid chromatography (RP-UPLC). Q = ‘Quarta’, R = ‘Record’, F = flower, SB = small berry, BB = big berry, PP = positive mode, PN = negative mode. *(Excel file)*

**Supplementary Table S6.** Metabolites A–Y among the top 50 metabolites (based on peak intensities) detected by metabolomic analysis. | = Separator for metabolites with more than one name. Classes not present in the PubChem, KEGG, ChEBI, MolGenie, LOTUS or LIPID MAPS databases are shown with the symbol (–).

| **Metabolites** | | **Class** |
| --- | --- | --- |
| (A) | (1r,3R,4s,5S)-4-{[(2E)-3-(3,4-dihydroxyphenyl)-2-propenoyl]oxy}-1,3,5-trihydroxycyclohexanecarboxylic acid | Organooxygen compounds |
| (B) | 3-[6-[[3,5-dihydroxy-6-methyl-4-[3,4,5-trihydroxy-6-(hydroxymethyl)oxan-2-yl]oxyoxan-2-yl]oxymethyl]-3,4,5-trihydroxyoxan-2-yl]oxy-2-(3,4-dihydroxyphenyl)-5,7-dihydroxychromen-4-one | Flavonoids |
| (C) | (3R,5R)-1,3,5-trihydroxy-4-[(E)-3-(4-hydroxyphenyl)prop-2-enoyl]oxycyclohexane-1-carboxylic acid | Organooxygen compounds |
| (D) | 3-[3,4-dihydroxy-6-(hydroxymethyl)-5-[3,4,5-trihydroxy-6-(hydroxymethyl)oxan-2-yl]oxyoxan-2-yl]oxy-2-(3,4-dihydroxyphenyl)-5,7-dihydroxychromen-4-one | Flavonoids |
| (E) | 2-(3,4-dihydroxyphenyl)-3-[(2S,3R,4S,5R,6R)-4,5-dihydroxy-3-[(2R,3R,4R,5R,6S)-3,4,5-trihydroxy-6-methyloxan-2-yl]oxy-6-[[(2R,3R,4R,5R,6S)-3,4,5-trihydroxy-6-methyloxan-2-yl]oxymethyl]oxan-2-yl]oxy-5,7-dihydroxychromen-4-one | Flavonoids |
| (F) | 5,7-dihydroxy-2-(4-hydroxyphenyl)-3-[(2S,3R,4S,5S,6R)-3,4,5-trihydroxy-6-[[(2R,3R,4R,5R,6S)-3,4,5-trihydroxy-6-methyloxan-2-yl]oxymethyl]oxan-2-yl]oxychromen-4-one | Flavonoids |
| (G) | 5-(β-d-glucopyranosyloxy)-2-hydroxybenzoic acid | – |
| (H) | l-methionine methylsulfonium | Carboxylic acids and derivatives |
| (I) | (S)-piperidine-2-carboxylic acid | Carboxylic acids and derivatives |
| (J) | (3E)-4-[(1S)-1-hydroxy-2,6,6-trimethyl-4-oxo-2-cyclohexen-1-yl]-3-buten-2-yl-β-D-glucopyranoside | – |
| (K) | (1S,3R,4S,5R)-1,3,5-trihydroxy-4-{[(2E)-3-(4-hydroxy-3-methoxyphenyl)-2-propenoyl]oxy}cyclohexanecarboxylic acid | – |
| (L) | 2-(3,4-dihydroxyphenyl)-5-hydroxy-4-oxo-4H-chromen-7-yl 2-O-(6-deoxy-α-l-mannopyranosyl)hexopyranoside | – |
| (M) | 3,5-dihydroxy-2-(4-hydroxyphenyl)-4-oxo-3,4-dihydro-2H-chromen-7-yl-hexopyranoside | – |
| (N) | (2S,3S,4S,5R,6R)-6-(3-benzoyloxy-2-hydroxypropoxy)-3,4,5-trihydroxyoxane-2-carboxylic acid | Organooxygen compounds |
| (O) | 4,8-dihydroxy-1-naphthyl β-d-glucopyranoside | – |
| (P) | 2,6-dihydroxy-2-[(4-hydroxyphenyl)methyl]-4-[(2S,3R,4S,5S,6R)-3,4,5-trihydroxy-6-(hydroxymethyl)oxan-2-yl]oxy-1-benzofuran-3-one | Flavonoids (Aurone) |
| (Q) | 2-[(1R,2R)-3-oxo-2-[(Z)-5-[3,4,5-trihydroxy-6-(hydroxymethyl)oxan-2-yl]oxypent-2-enyl]cyclopentyl]acetic acid | Fatty acyls |
| (R) | (1S,3R,4R,5R)-3,4-bis[[(E)-3-(3,4-dihydroxyphenyl)prop-2-enoyl]oxy]-1,5-dihydroxycyclohexane-1-carboxylic acid | Organooxygen compounds |
| (S) | methyl (1S,3R,4R,5R)-3-[(E)-3-(3,4-dihydroxyphenyl)prop-2-enoyl]oxy-1,4,5-trihydroxycyclohexane-1-carboxylate | Organooxygen compounds |
| (T) | 3,5,5-trimethyl-4-[3-[(2R,3R,4S,5S,6R)-3,4,5-trihydroxy-6-(hydroxymethyl)oxan-2-yl]oxybutyl]cyclohex-2-en-1-one\|4-(2,6,6-trimethyl-4-oxo-2-cyclohexen-1-yl)-2-butanyl β-d-glucopyranoside | – |
| (U) | 5′-deoxy-5′-(methylthio)adenosine | 5'-deoxyribonucleosides |
| (V) | 3-deoxy-d-manno-2-octulosonic acid | Organooxygen compounds |
| (X) | 2-(3,4-dihydroxyphenyl)-3-[(2S,3R,4S,5S,6R)-4,5-dihydroxy-3-[(2R,3R,4R,5R,6S)-3,4,5-trihydroxy-6-methyloxan-2-yl]oxy-6-[[(2R,3R,4R,5R,6S)-3,4,5-trihydroxy-6-methyloxan-2-yl]oxymethyl]oxan-2-yl]oxy-5,7-dihydroxychromen-4-one | Flavonoids |
| (Y) | methyl 2-[(2S,3R,4S,5S,6R)-3,4,5-trihydroxy-6-[[(2S,3R,4S,5R)-3,4,5-trihydroxyoxan-2-yl]oxymethyl]oxan-2-yl]oxybenzoate | Organooxygen compounds |

**Supplementary Table S7.** Kallisto mapping (%). F = flower, SB = small berry, BB = big berry.

| **Cultivar** | **Tissue** | **Sample** | **Mapping (%)** |
| --- | --- | --- | --- |
| ‘Quarta’ | Flower | QF1 | 81.6 |
|  |  | QF3 | 76.9 |
|  |  | QF5 | 77.9 |
|  | Small berry | QSB1 | 73.9 |
|  |  | QSB3 | 69.6 |
|  |  | QSB5 | 75.0 |
|  | Big berry | QBB1 | 75.6 |
|  |  | QBB3 | 74.6 |
|  |  | QBB5 | 74.8 |
| ‘Record’ | Flower | RF1 | 78.4 |
|  |  | RF2 | 77.6 |
|  |  | RF3 | 76.5 |
|  | Small berry | RSB2 | 75.6 |
|  |  | RSB3 | 75.3 |
|  |  | RSB4 | 74.8 |

**Supplementary Table S8.** Transcripts per million (TPM) from transcripts across all RNA-Seq samples. Q = ‘Quarta’, R = ‘Record’, F = flower, SB = small berry, BB = big berry. *(Excel file)*

**Supplementary Table S9.** Gene lists obtained when comparing different sample types, assembled using Venny 2.1.0 for upregulated and downregulated genes. *(Excel file)*

**Supplementary Table S10.** MapMan4 categories obtained from enrichment analysis (one-sided Fisher’s exact test, over-representation analysis, FDR < 0.05) of upregulated and downregulated transcripts. *(Excel file)*

**Supplementary Figure S5.** Enrichment analysis of (a) upregulated and (b) downregulated transcripts between QF × QSB, QF × QBB, and QSB × QBB. Significantly enriched terms (FDR < 0.05) are displayed (one-sided Fisher’s exact test, over-representation analysis). The top 30 significantly enriched terms (FDR < 0.05) are displayed. Bubble size corresponds to the number of identified transcripts per term, with color indicating adjusted p-values transformed using -log10. Q = ‘Quarta’, R = ‘Record’, F = flower, SB = small berry, BB = big berry.

**Supplementary Figure S5, continued.** Enrichment analysis of (a) upregulated and (b) downregulated transcripts between QF × QSB, QF × QBB, and QSB × QBB. Significantly enriched terms (FDR < 0.05) are displayed (one-sided Fisher’s exact test, over-representation analysis). The top 30 significantly enriched terms (FDR < 0.05) are displayed. Bubble size corresponds to the number of identified transcripts per term, with color indicating adjusted p-values transformed using -log10. Q = ‘Quarta’, F = flower, SB = small berry, BB = big berry.

**Supplementary Table S11.** Genes within the “secondary metabolism” MapMan4 category and their corresponding functions. (*Excel file*)

**Supplementary Table S12.** IDs from genes described in phenylpropanoid (Figure 8) and terpenoids and cholesterol (steroid) (Figure 9) pathways. *PAL*, phenylalanine ammonia lyase; *C4H*: cinnamic acid 4-hydroxylase; *4CL*, 4-coumarate-CoA ligase; *C3′H*, *p*-coumaroyl quinate/shikimate 3′-hydroxylase; *HCT*, hydroxycinnamoyl CoA shikimate/quinate:hydroxycinnamoyl transferase; *HQT*, hydroxycinnamoyl CoA quinate:hydroxycinnamoyl transferase; *CHS*, chalcone synthase; *CHI*, chalcone isomerase; *FNS*, flavone-synthase; *F3H*, flavanone-3-hydroxylase; *F3′H*, flavonoid-3′-hydroxylase; *FLS*, flavonol synthase; *3GT*, flavonoid 3-*O*-glucosyltransferase; *RT*, 3-*O*-rhamnosyltransferase; *F3GalTase*, kaempferol 3-*O*-β-d-galactosyltransferase; *DXS*, 1-deoxy-d-xylulose 5-phosphate synthase; *CMK*, 4-diphosphocytidyl-2-C-methyl-d-erythritol kinase; *GGPS*, geranylgeranyl pyrophosphate synthase; *GGPS-LSU*, geranyl diphosphate synthase large subunit; *HMGCS*, HMG-CoA synthase; *HMGCR*, 3-hydroxy-3-methylglutaryl-coenzyme A reductase; *SQS*, squalene synthase; *SQLE*, squalene epoxidase; *SM*, squalene monooxygenase. (*Excel file*)
